# Silencing the G-protein coupled receptor 3-salt inducible kinase 2 pathway promotes human β cell proliferation

**DOI:** 10.1038/s42003-021-02433-2

**Published:** 2021-07-23

**Authors:** Caterina Iorio, Jillian L. Rourke, Lisa Wells, Jun-Ichi Sakamaki, Emily Moon, Queenie Hu, Tatsuya Kin, Robert A. Screaton

**Affiliations:** 1 Sunnybrook Research Institute, Toronto, Canada; 2 Department of Biochemistry and Molecular Biology, Graduate School of Medicine, The University of Tokyo, Bunkyo-ku, Japan; 3 Department of Biochemistry, University of Toronto, Toronto, Canada; 4 Clinical Islet Laboratory, University of Alberta Hospital, Edmonton, Canada; 5 Present Address: Mount Allison University, Sackville, NB Canada

**Keywords:** Cell-cycle exit, Type 1 diabetes

## Abstract

Loss of pancreatic β cells is the hallmark of type 1 diabetes, for which provision of insulin is the standard of care. While regenerative and stem cell therapies hold the promise of generating single-source or host-matched tissue to obviate immune-mediated complications, these will still require surgical intervention and immunosuppression. Here we report the development of a high-throughput RNAi screening approach to identify upstream pathways that regulate adult human β cell quiescence and demonstrate in a screen of the GPCRome that silencing G-protein coupled receptor 3 (GPR3) leads to human pancreatic β cell proliferation. Loss of GPR3 leads to activation of Salt Inducible Kinase 2 (SIK2), which is necessary and sufficient to drive cell cycle entry, increase β cell mass, and enhance insulin secretion in mice. Taken together, our data show that targeting the GPR3-SIK2 pathway is a potential strategy to stimulate the regeneration of β cells.

## Abstract (web summary)

Caterina Iorio et al. use a high-throughput RNAi screen to identify the GPR3-SIK2-CDKN1B axis as key players involved in regulating pancreatic beta cell proliferation. These results suggest that GPR3 and SIK2 are both potential targets to stimulate regeneration of beta cells in diseases like type 1 diabetes.

## Introduction

Progress in understanding human β cell proliferative behavior has been hampered by the human β cell being a comparatively intractable system due to scarcity of tissue and a lack of genetic means to study them. Consequently, our knowledge of mechanisms of β cell proliferation comes largely from studies in rodents, where adult β cell proliferation is readily observed. As β cells are among the longest-lived cells in the mouse, mouse and human β cells share at least some degree of proliferative intransigence^1–5^. However, key species differences in the cell cycle proteome^6–10^ have necessitated research into human β cell biology. While phenotypic HTS of small molecule libraries is an efficient way to identify lead compounds that elicit human β cell proliferation to meet translational goals, a lack of understanding of the target and the mechanism of action of small molecule mitogens, as well as their off-target effects, remain a major impediment^11^.

We have argued that a safe regenerative approach will first require a genetic dissection of complex regulatory mechanisms governing the stable quiescence of human adult β cells^12^; understanding the genetic program that establishes and maintains this quiescence is of both biological interest and therapeutic promise. In a small-scale RNAi screen targeting cell cycle components, we previously reported that ~10% of human β cells enter the cell cycle following silencing of the cyclin-dependent kinase inhibitors (CDKIs) CDKN2C/p18 or CDKN1A/p21^12^. Here, we scale this approach to make it compatible with automation and use it to interrogate an RNAi library targeting the human G protein-coupled receptor (GPCR) family to identify regulators of human β cell proliferation.

## Results

### High-throughput RNAi screen in primary human islet cells

**Fig. 1:**
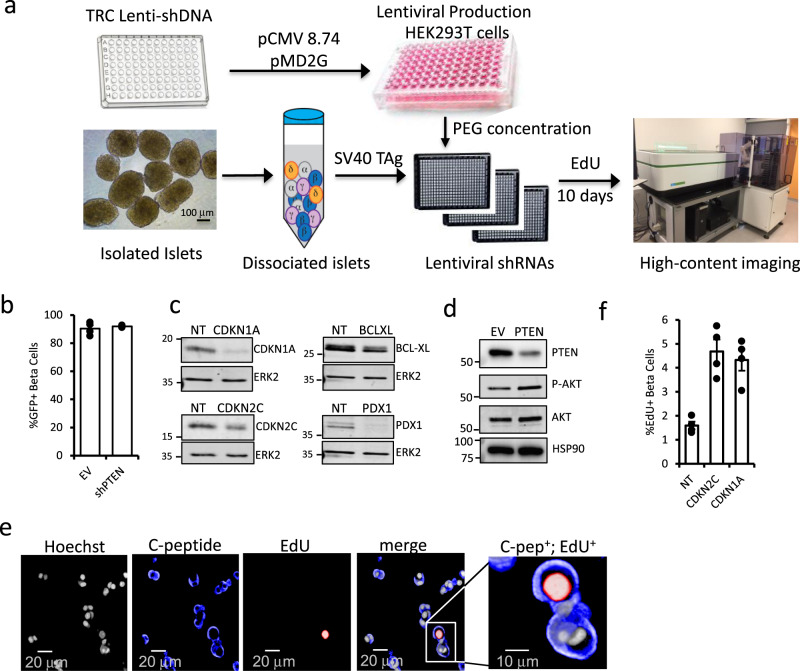
Automated HTS assay to screen in primary human cells. **a** Schematic showing the workflow of HTS screen, from shDNA plasmid packaging into lentivirus, arraying into 384-well plates and seeding with dissociated human islet cells, prior to 10d culture to permit gene silencing and EdU incorporation. **b** Bar plot showing % GFP positive, C-peptide positive cells after infection with HTS virus encoding shRNA targeting PTEN or empty vector control. **c** Representative Western blots showing knockdown efficiency of positive controls following infection with lentiviral-shRNAs against CDKN2C/p18, CDKN1A/p21, BCL-XL, and PDX1. **d** Effect of silencing PTEN on downstream effector phospho-AKT, with total AKT and HSP90 control is shown. **e** Representative images of dissociated human islets from positive control well coinfected with TAg and silenced for CDKN1A then stained for nuclei (Draq5, gray), C-peptide (blue), EdU (red), and merged. Scale bar = 20 µm. Zoom merged image is shown (Scale bar = 10 µm). **f** Barplot confirming the effectiveness of lentivirus prepared with HTS protocol on silencing CDKN2C and CDKN1A and stimulating human β cell proliferation. *p*-values: CDKN2C = 0.0011 and CDKN1A = 0.0012. Error bars are defined as the standard error of the mean for *n* = 4 technical replicates for all statistics. MWs for Western blot markers are in kDa.

To make our lentiviral silencing approach scalable for genome-wide screens while remaining compatible with screening in primary human tissue (Fig. 1a), we developed a robotics-compatible HTS protocol, starting from shDNA plasmid isolation through to virus purification and target cell infection. This approach generated lentivirus achieving >90% infection of adult human β cells (Fig. 1b), which maintain their identity following infection over the 10-day assay time course (Supplementary Fig. 1). We observed effective reduction of several target proteins with HTS virus, including PDX1, PTEN, CDKN1A, CDKN2C, and BCL-XL (Fig. 1c). When PTEN was silenced, we observed an increase in phosphorylation of the PTEN target AKT at Ser473 (Fig. 1d), confirming knockdown and that expected functional consequences are preserved following infection with HTS virus. For over 40 years, cell cycle analysis has relied upon DNA tumor viruses such as adenovirus and SV40 to identify critical cell cycle components, including the tumor suppressors p53 and pRb^13^. Thus, we primed β cells to proliferate using SV40 large T antigen (TAg) and stained for incorporation of the S-phase marker EdU in C-peptide+ cells (Fig. 1e). Following silencing of control targets CDKN2C or CDKN1A with HTS virus, we observed a threefold increase in EdU+ C-peptide+ cells over non-targeting (NT) shRNA (Fig. 1f). Under these conditions, <5% of C-peptide+ cells stain for activated caspase-3, and EdU+ cells are not caspase-3-positive (Supplementary Fig. 2).

### Silencing GPR3 promotes human β cell proliferation

**Fig. 2:**
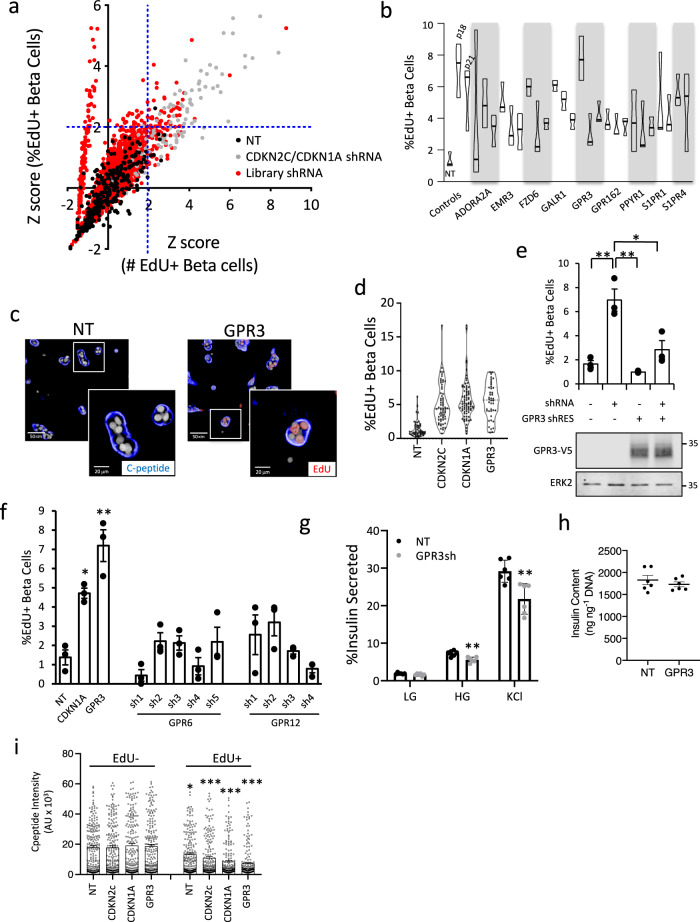
GPCRome screen identifies GPR3 as a regulator of human β cell proliferation. **a** Z-score plot of screen results. Control cells infected with non-targeting virus (black circles, NT), positive control CDKN2C or CDKN1A shRNA virus (gray circles), or GPCRome library shRNAs (red circles) are shown. The *x*-axis represents Z-scores of double-positive (EdU, insulin) cells based on the absolute number; the *y*-axis represents Z-scores for the percentage of total b cells. Dotted blue lines mark the threshold of Z-scores = 2. **b** Validation data for candidates identified in the screen. Percentage proliferation achieved for individual shRNAs against indicated targets was averaged over three screens (*n* = 3 technical replicates/donor) using islets from three independent donors. **c** Screen images showing representative NT control or GPR3 shRNA-infected b cells (C-peptide, blue) stained for EdU (red). **d** Violin frequency distribution plots showing the percentage of EdU+ insulin+ cells following GPR3 silencing in 18 donors over a 10-day assay period compared to non-targeting control (NT) and CDKN2C and CDKN1A positive controls. Dark line = median, light line = quartiles. **e** Barplot showing the percentage of EdU+ β cells following silencing GPR3 in the presence of the shRNA-resistant GPR3 cDNA tagged with V5 (GPR3-RES-V5) or with NT shRNA control. Western blots showing expression of exogenous GPR3 are shown underneath. **f** Barplot showing the percentage of EdU+ β cells following silencing of GPR3 and five independent shRNAs targeting GPR6 and four independent shRNAs targeting GPR12, the most closely related GPCRs to GPR3. **g** GSIS in cells silenced for GPR3 compared to NT control. LG low glucose, HG high glucose, KCl depolarizing stimulus. *p*-values for GPR3 HG = 0.002; GPR3 KCl = 0.005. n = 6 technical replicates. **h** Insulin content in cells silenced for GPR3 compared to NT control. *n* = 6 technical replicates. **i** Dotplot showing C-peptide intensity in EdU+ and EdU− populations in cultures silenced for indicated targets. Non-targeting (NT) control shown. *n* = 200 β cells per condition. All cultures were infected with TAg. *p*-values: NT = 0.0012; CDKN2C = 1.4 × 10^−14^; CDKN1A = 5.0 × 10^−6^; GPR3 = 3.4 × 10^−12^. All plots are representative of three biological replicates. Error bars are defined as the standard error of the mean for *n* = 3 technical replicates unless stated otherwise. MWs for Western blot markers are in kDa.

Human islets express numerous GPCRs^14,15^ which govern a myriad of key islet functions from hormone production and secretion to proliferation^16–18^ and are important targets for diabetes treatment^19–21^. The relatively low abundance of GPCR mRNA and protein permit efficient knockdown for RNAi screens and their druggability make them attractive targets for translation^22,23^. Therefore, we elected to silence 397 GPCRs and 40 of their cytoplasmic adaptors, the “GPCRome”, to identify GPCR signaling pathways that promote human β cell quiescence (see Supplementary Data 2 for list of genes and shRNA sequences). We pooled 450,000 freshly isolated glucose-responsive islet equivalents from two non-diabetic donors (Supplementary Fig. 3). Following dissociation, islet cells were seeded in 384-well plates containing 2342 pre-arrayed lentivirus encoding SV40 TAg together with individual library lenti-shRNAs, then cultured for 10 days in the presence of EdU. Z-scores for both the absolute number and percentage of C-peptide+ cells that also stained positive for EdU were determined (Fig. 2a).

Nine candidates with at least two shRNAs with Z-scores ≥2 for both criteria were evaluated in confirmation screens using islets from three independent donors (Fig. 2b). Of the candidates identified, G-protein-coupled receptor 3 (GPR3) has been implicated in cell cycle arrest and survival signaling in cerebellar development^24,25^, and in germ cell cycle arrest in *X. Laevis*^26^ and mammalian oocytes^27–29^, so we selected GPR3 for validation. Visual analysis of GPR3 silenced cultures revealed the presence of numerous EdU+ cells and even nuclear doublets (Fig. 2c), suggestive of mitotic events. Silencing GPR3 mRNA (Supplementary Fig. 4) gave significant increases in EdU incorporation in β cells in all subsequent donors tested (*n* = 18, Fig. 2d). To rule out off-target effects of the GPR3 shRNA, we expressed an shRNA-resistant GPR3 cDNA to reconstitute GPR3 protein, which restored quiescence in the presence of GPR3 shRNA and reduced basal proliferation when expressed with control shRNA (Fig. 2e). Silencing GPR3’s closest phylogenetic neighbors, GPR6 and GPR12^30^, did not induce proliferation above baseline, indicating a specific role for GPR3 in maintaining cell cycle arrest (Fig. 2f). The silencing of GPR3 also increased β cell proliferation in the absence of TAg (Supplementary Fig. 5). Taken together, we conclude that silencing GPR3 can reverse the stable quiescence of adult human β cells. Knockdown of GPR3 in dispersed and intact islets had a negligible effect on glucose-stimulated insulin secretion (GSIS) (Fig. 2g) or insulin content (Fig. 2h), showing β cells lacking GPR3 maintain functionality. In contrast, the intensity of C-peptide staining in EdU+ cells was 25–70% lower than in EdU- cells (Fig. 2i), consistent with previous observations documenting downregulation of insulin expression in proliferating cells^12^.

### GPR3 promotes cell cycle arrest by stabilizing CDKN1A and CDKN1B

**Fig. 3:**
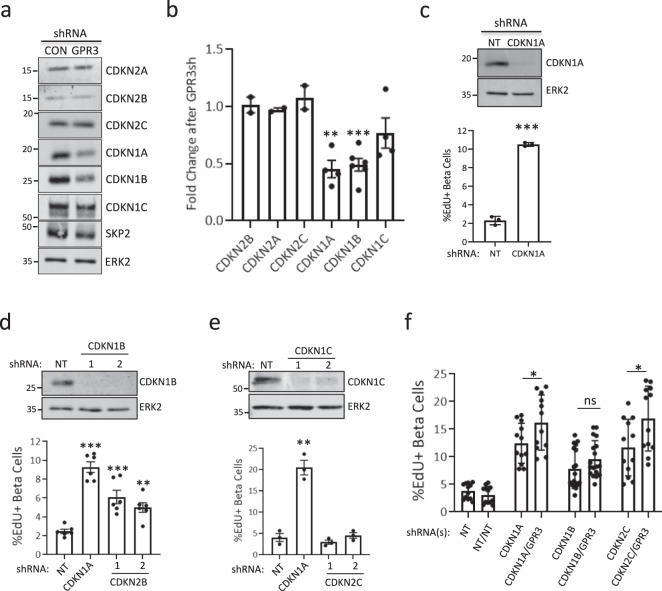
GPR3 stabilizes CIP/KIP cell cycle-dependent kinase inhibitors. **a** Representative Western blot analysis of INK and CIP/KIP family CKI levels in NT control and GPR3-silenced human islet cells. *n* = 3 biological replicates. **b** Bar plot showing quantitation of CKI intensities. The *p*-value for CDKN1A = 0.0003; CDKN1B = 3.5 × 10^−6^. Error bars are defined as the standard error of the mean of at least three biological replicates. **c** Western blot and barplot of human β cell proliferation following silencing of CDKN1A. *p*-value = 7.0 × 10^−6^. **d** Western blot and barplot of human β cell proliferation following silencing of CDKN1B; *p*-values = 0.0005 and 0.001. CDKN1A control *p*-value = 4.1 × 10^−7^. **e** Western blot and barplot of human β cell proliferation following silencing of CDKN1C. CDKN1A control *p*-value = 0.001. All samples co-express TAg. For (**c**)–(**e**), blots and bar plots are representative of two biological replicates. Error bars are defined as the standard error of the mean of three technical replicates. **f** Barplot showing human β cell proliferation following silencing of CDKN2C, CDKN1A, or CDKN1B alone or in combination with GPR3. All samples co-express TAg. No virus added is indicated by a dash. NT non-targeting shRNA control. The error bars are defined as the standard error of the mean of three biological replicates. MWs for Western blot markers are in kDa.

We previously reported that silencing the CDKN2C/p18 and CDKN1A/p21 in human β cells leads to S-phase entry^12^. We reasoned that silencing GPR3 would reduce levels of one or more CDKIs, so we performed Western blots for the INK4 class (CDKN2A/p16, CDKN2B/p15, CDKN2C) and the more broadly acting CIP/KIP class (CDKN1A, CDKN1B, and CDKN1C/p57) of CDKIs. Of these, only CDKN1A and CDKN1B of the CIP/KIP class of CDKIs were reduced following GPR3 silencing (Fig. 3a, b); the CIP/KIPs CDKIs inhibit the assembly and catalytic activity of Cyclin D-dependent CDK complexes in the G0-G1 transition, Cyclin E-CDK2 complexes in G1, and Cyclin A-CDK2 complexes in the S to G2 transition^31^. The CIP/KIP class is turned over by an SCF ubiquitin ligase complex that harbors the F-box protein SKP2 as its substrate-selectivity component^32^, raising the possibility that GPR3 signaling may reduce SKP2 levels, and in turn, increase levels of CIP/KIPs. Unexpectedly, SKP2 levels were not increased following GPR3 silencing, indicating that accumulation of SKP2 does not mediate the loss of CIP/KIP proteins in this context (Fig. 3a). Consistent with our previous work, silencing CDKN1A induced β cell cycle entry (Fig. 3c). To determine if loss of CDKN1B or CDKN1C alone is sufficient for the induction of proliferation, we silenced each with shRNAs and observed proliferation following CDKN1B^33^ (Fig. 3d), but not CDKN1C^34^ (Fig. 3e) silencing. To determine if GPR3 and the CDKIs CDKN2C^35^, CDKN1A, and CDKN1B work in the same pathway, we performed co-silencing experiments to explore possible epistatic relationships between GPR3 and the CDKIs (Fig. 3f). Whereas co-silencing CDKN2C or CDKN1A with GPR3 resulted in enhanced proliferation over the CDKIs alone, silencing GPR3 together with CDKN1B did not. Taken together, our data suggest that CDKN2C, CDKN1A, and CDKN1B are the key CDKIs that prevent proliferative activity in adult human β cells and that GPR3 promotes cell cycle arrest by maintaining levels of CDKN1A and CDKN1B.

### The GPR3-SIK pathway maintains human β cell quiescence

**Fig. 4:**
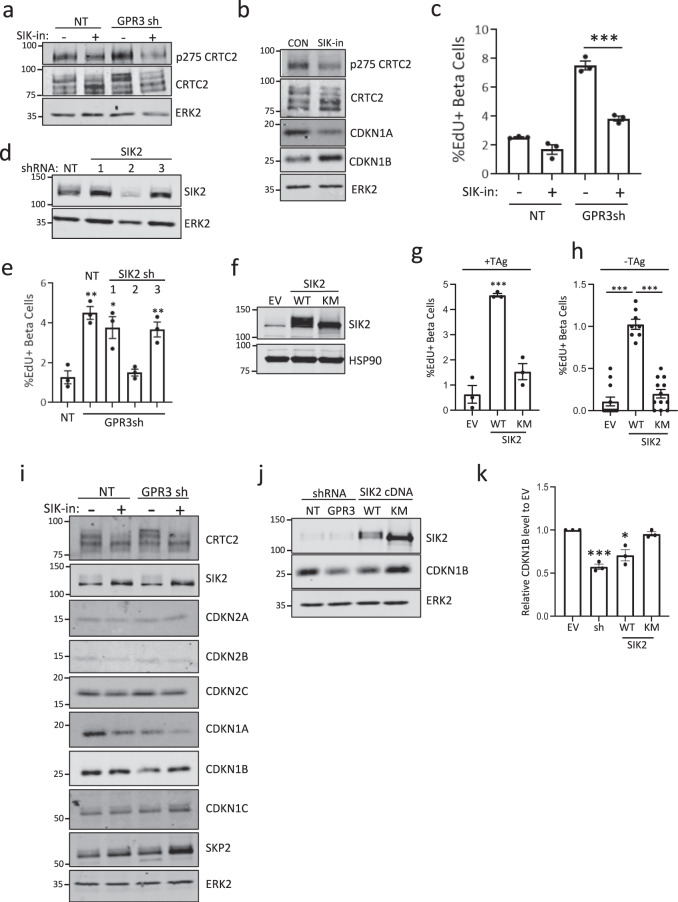
GPR3 promotes quiescence by inhibiting SIK2. **a** Western blot showing an increase in the phosphorylation status of SIK2 target protein CRTC2. The effect of pan-SIK inhibitor (SIK-in) is shown. **b** Western blot showing the effect of SIK-in on levels of CDKN1A and CDKN2B in human islet cells. **c** Barplot showing the effect of SIK-in on proliferation in GPR3-silenced human β cells. *p*-value = 0.00047. **d** Western blots showing silencing of SIK2 with shRNA2. **e** Effect of co-silencing SIK2 in cells silenced for GPR3 on β cell proliferation. *p*-values vs NT: GPR3sh = 0.0021, GPR3sh + SIK2sh1 = 0.018, GPR3sh + SIK2sh3 = 0.009. **f** Western blot showing levels of SIK2 wild type (WT) and kinase-dead (KM mutant) proteins. **g**, **h** Barplots showing the effect of overexpression of SIK2 WT and kinase-dead (KM) mutants of SIK2 on β cell proliferation in the presence and absence of TAg. EV vs. KM = not significant. The *p*-value for *g* = 0.00042. *p*-values for (**h**) are 3.35 × 10^−9^ and 1.35 × 10^−8^. **i** Western blots showing levels of CIP/KIP proteins in GPR3 silenced β cells. Effect of pan-SIK inhibitor HG-9-91-01 (SIK-in) shown. **j** Western blots showing CDKN1B levels in cells following GPR3 silencing or overexpression of SIK2 WT or kinase-dead mutant (KM). **k** Barplot showing quantification of CDKN1B levels. *p*-values are 0.0002 and 0.0103. Error bars are defined as the standard error of the mean of three biological replicates. All western blots are representative blots from three donors. For (**c**), (**e**), (**g**), and (**h**), the error bars are representative of three donors and are defined as the standard error of the mean of three technical replicates. MWs for Western blot markers are in kDa.

GPR3 promotes constitutive cAMP synthesis through adenylyl cyclase via an unknown ligand^27^ which prompted us to evaluate the role of PKA signaling in GPR3-mediated cell cycle arrest^36,37^. We monitored the phosphorylation status of the transcriptional coactivator CRTC2, which becomes dephosphorylated when PKA is active^36^. Western blots of cells silenced for GPR3 show that CRTC2 became more phosphorylated, consistent with inhibition of CRTC2 and reduced PKA activity (Fig. 4a). As Ser275 on CRTC2 is a target of salt inducible kinase 2 (SIK2)^37,38^, we treated islets with the pan-SIK inhibitor HG-9-91-01 (SIK-in), which led to CRTC2 dephosphorylation (Fig. 4b) and restored quiescence in GPR3-silenced cells (Fig. 4c). Taken together, we conclude that GPR3 promotes quiescence in the adult β cell by inhibiting SIKs.

As SIK-in inhibits SIK1, SIK2, and SIK3, we next used RNAi to identify which SIKs are required for GPR3 to maintain β cell quiescence. When GPR3 was silenced together with each SIK, proliferation rates reversed back to control levels only when SIK2 was co-silenced with GPR3 (Fig. 4d, e), silencing SIK1 and SIK3 had no effect (Supplementary Fig. 6). Consistent with a requirement for SIK2 for β cell proliferation, overexpression of a wild type SIK2, but not a kinase-dead SIK2 mutant (KM; Fig. 4f), was able to increase proliferation rates to those observed in GPR3-silenced cells both in the presence (Fig. 4g) and absence of TAg (Fig. 4h). To determine which CDKIs are regulated by SIKs, we treated control and GPR3-silenced cultures with SIK-in and performed Western blots. Whereas levels of CDKN1A and CDKN1B are reduced by GPR3 silencing, only levels of CDKN1B protein were restored following SIK inhibition (Fig. 4i). Consistent with this, overexpression of SIK2 in human islets, but not SIK2-KM (Fig. 4j), reduced levels of CDKN1B as effectively as silencing GPR3 (Fig. 4k).

### SIK2 promotes β cell proliferation in vivo

**Fig. 5:**
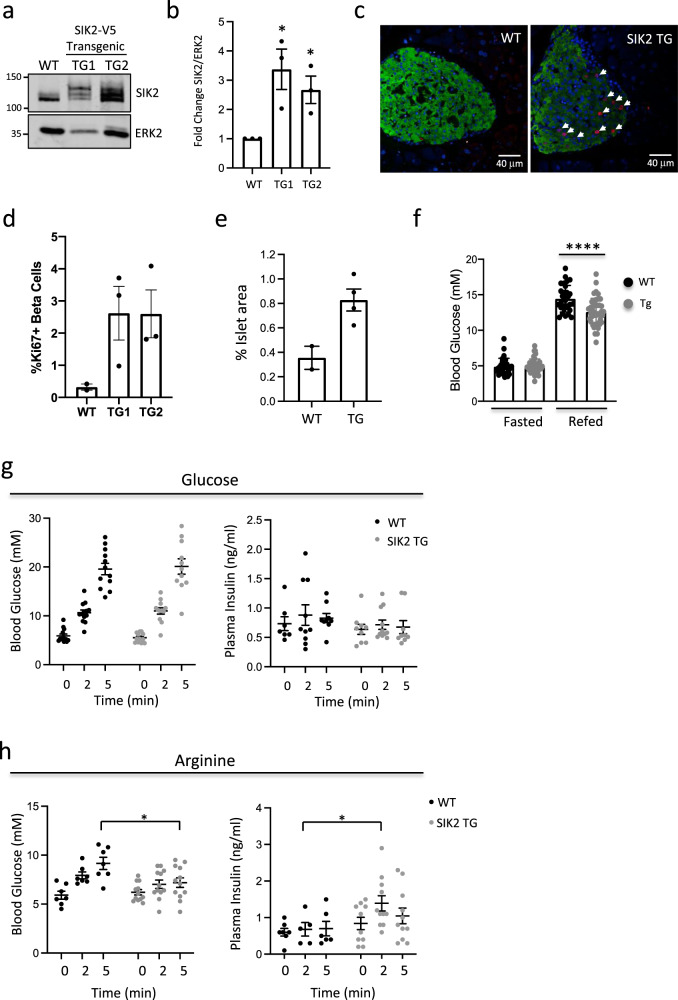
SIK2 promotes mouse β cell proliferation. **a** Western blot showing levels of endogenous and SIK2-V5 protein in control and MIP-SIK2 transgenic (SIK2-V5-TG) animals with an ERK2 loading control. **b** Bar plot showing fold change in SIK2 protein in SIK2 TG1 and TG2 relative to WT (10–14 weeks). *p*-values = 0.0272 and 0.0245. Error bars are defined as the standard error of the mean of three biological replicates. **c** Ki67 (red) immunostaining of pancreatic sections from female WT (left) and SIK2 Tg mice (12–16 weeks). Arrowheads show proliferating β cells. Hoechst staining of nuclei shown (blue). **d** Barplot showing quantification of Ki67+ insulin+ cells from female WT and SIK2 transgenic founders 1 and 2. In total, 1000–1500 cells were counted for each. Error bars are defined as the standard error of the mean of three biological replicates. **e** Barplot showing % islet area in WT and SIK2 Tg mice. *p-*value = 0.0322. Error bars are defined as the standard error of the mean of three biological replicates. **f** Barplot showing fasting and fed blood glucose concentrations for WT and founder line 2 of MIP-SIK2 (SIK2 TG) mice at 16 weeks of age. *p*-value = 0.00011. Error bars are defined as the standard error of the mean of 10 biological replicates for WT and 15 biological replicates for TG. **g** Scatter charts showing blood glucose and plasma insulin in male and female WT (black dots) and SIK2 Tg (gray dots) mice (8–16-weeks) following IP injection of glucose. Error bars are defined as the standard error of the mean of 12(WT)/11(TG) biological replicates for blood glucose and 7(WT)/9(TG) biological replicates for plasma insulin data. **h** Scatter charts showing blood glucose and plasma insulin in male and female WT (black dots) and SIK2 Tg (gray dots) mice (8–16-weeks) following IP injection of arginine. *p*-values are 0.025 and 0.049. Error bars are defined as the standard error of the mean of 7(WT)/12(TG) biological replicates for blood glucose and 5(WT)/10(TG) biological replicates for plasma insulin data. MWs for Western blot markers are in kDa.

Deregulated SIK2 has been reported to support proliferative behavior in other contexts^39^, thus we postulated that enhanced levels of SIK2 may enhance proliferation of β cells in vivo. To address this, we generated SIK2 (MIP-SIK2-V5) transgenic (Tg) founder mice that expressed approximately two to fourfold higher SIK2 in isolated islets than control mice (Fig. 5a, b; Supplementary Fig. 7), levels similar to those we observed in non-diabetic ob/ob or Western diet-fed mice^37^. Ki67 staining (Fig. 5c) revealed that elevated SIK2 expression was sufficient to increase average proliferation rates from 0.3% to over 2% (Fig. 5d) and increase islet area twofold (Fig. 5e). At 16–20 weeks, MIP-SIK2 Tg mice display normal fasting blood glucose and normal glucose tolerance (Supplementary Fig. 8) but reduced blood glucose levels after 1 h refeeding (Fig. 5f). Interestingly, these SIK2 Tg mice show no change in responsiveness to acute IP glucose challenge (Fig. 5g), but show significantly higher basal insulin secretion and after arginine challenge (Fig. 5h), characteristic of a fetal β cell functional profile^40^.

### Elevated SIK2 and reduced CDKN1B are hallmarks of β cell compensation in humans

**Fig. 6:**
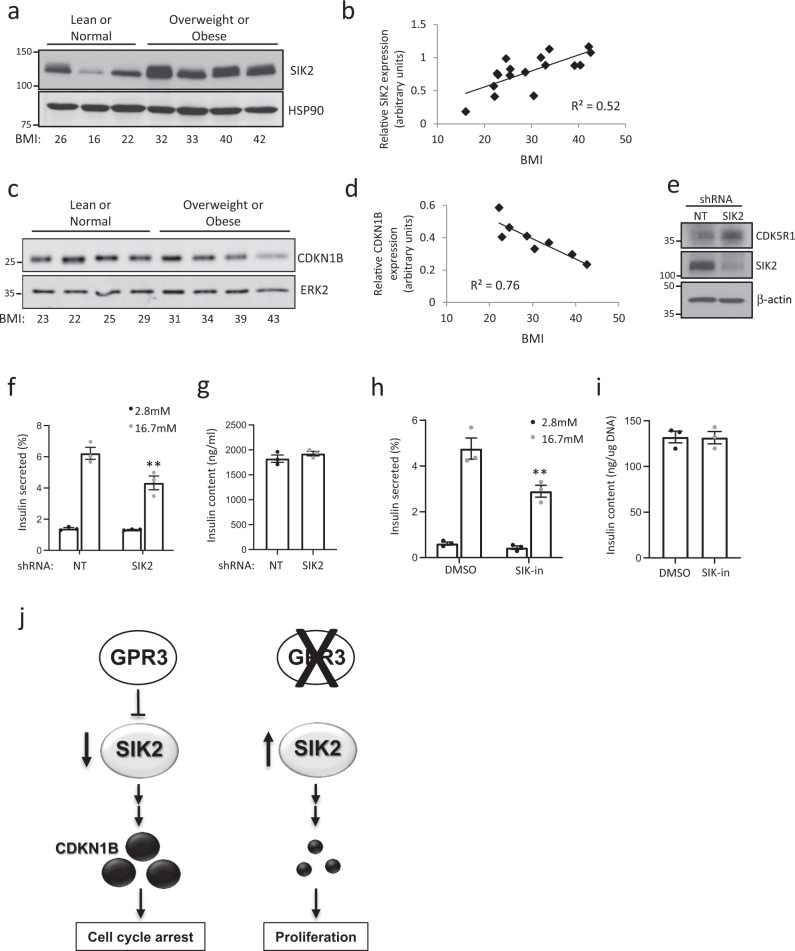
SIK2 promotes human β cell function. **a** Western blots showing levels of SIK2 protein and HSP90 loading control in isolated human islets from non-diabetic human subjects of increasing BMI. **b** Line plot showing a correlation between SIK2 levels in isolated human islets and subject’s BMI; *r*^2^ = 0.52. **c** Western blots showing levels of CDKN1B protein and ERK2 loading control in isolated human islets from non-diabetic human subjects of increasing BMI. **d** Line plot showing a correlation between SIK2 levels in isolated human islets and subject’s BMI; *r*^2^ = 0.76. **e** Western blot showing levels of SIK2 and its substrate CDK5R1/p35 following silencing of SIK2 compared to NT control. **f** Barplots showing insulin secretion in response to 16.7 mM glucose following silencing of SIK2 in isolated human islets compared to NT control. *p*-value = 0.0327. **g** Insulin content in islets shown in (**f**). **h** Barplot showing the effect of SIK-in on GSIS in isolated human islets compared to DMSO control. *p*-value = 0.0364. **i** Insulin content in islets shown in (**h**). For (**f**)–(**i**), bar plots are representative of *n* = 3 donors. For each plot, error bars are defined as the standard error of the mean of three technical replicates. **j** Model showing GPR3 promoting CDKN1B accumulation to maintain cell cycle arrest via inhibition of SIK2. When GPR3 is silenced, SIK2 becomes active and CDKN1B is lost, facilitating cell cycle entry. MWs for Western blot markers are in kDa.

Consistent with these data, SIK2 protein in islets isolated from non-diabetic human subjects ranging from lean to obese BMI (Fig. 6a) increased linearly (Fig. 6b, *r*^2^ = 0.52), supporting the notion that increased SIK2 in the human islet permits a compensatory secretory response. Levels of CDKN1B were reduced with increasing BMI, indicating that increased levels of SIK2 correlate with loss of CDKN1B in human subjects (Fig. 6c, d). Levels of the β cell marker PDX1 were unchanged (Supplementary Fig. 9). We previously described a complex consisting of SIK2, the CDK5 activator CDK5R1/p35, and the E3 ligase PJA2 that is essential for functional compensation in the β cell, in which SIK2 protein accumulates in islets of prediabetic mice^37^. In keeping with our animal data, silencing SIK2 in human islets resulted in increased levels of the SIK2 target p35 (Fig. 6e) and reduced glucose-stimulated insulin secretion (GSIS) by 35% (Fig. 6f) without affecting insulin content (Fig. 6g). Similarly, treatment with SIK-in to inhibit all SIKs reduced GSIS by 40% (Fig. 6h) without affecting insulin content (Fig. 6i), consistent with a specific role for SIK2 in this regard.

## Discussion

Thus, strategies designed to restore lost or damaged tissue must be subject to reversal and will require a complete understanding of the proliferative machinery and their regulation in the human β cell. Our work identifies GPR3 as a key cell surface receptor that suppresses SIK2 activity to stabilize CIP/KIP CDKIs and maintain quiescence. We found that silencing GPR3 in the presence of TAg triggered ~5–6% of primary human β cells to proliferate, which could be rescued by restoration of GPR3 with RNAi-resistant cDNA, establishing GPR3 as a *bona fide* target. Consistent with this, mild overexpression of GPR3 reduced proliferation in control cells, indicating that proliferative potential in human β cells is finely tuned to the levels of GPR3. That we do not achieve 100% proliferation with silencing CDKN1A, CDKN1B, CDKN2C, or GPR3 in the presence of TAg is consistent with our proposal that a complex genetic program consisting of multiple redundant anti-proliferative pathways is activated in the adult β cell to maintain a stable quiescence^12^. Of note, GPR3 silencing lowers the more promiscuous CIP/KIP CDKIs, demonstrating its key role as a regulator of β cell quiescence—defined as a withdrawal from the cell cycle, which may share some overlapping properties of G1/S and/or G2/M cell cycle arrest—and underscoring the likelihood that numerous roadblocks to β cell cycle entry must be overcome prior to entering S-phase. Owing to the ontological commonality between neurons and β cells, it is interesting that GPR3 also establishes quiescence of cerebellar granule neurons during postnatal development in mice^24^, raising the possibility of a general role for GPR3 in regulating cell cycle entry decisions in excitable cells. Indeed, silencing GPR3 also resulted in cell cycle entry in α and δ cells, suggesting it can serve a broader role in suppressing cell cycle entry of the neuroendocrine lineage (Supplementary Fig. 10). The GPR3 whole animal knockout results in age-dependent obesity and mild glucose intolerance due to impaired thermogenesis, but serum insulin and C-peptide levels were unchanged^41^. Extra-islet compensatory effects (brain, fat) in the GPR3 knockout will require clarification of GPR3’s role in vivo by tissue-specific deletion of GPR3. It is noteworthy that EdU-positive cells in all conditions show a reduction in C-peptide staining intensity, suggestive of loss of β cell functional identity in proliferating β cells^12^. This is in keeping with the idea that the fully mature β cell state and cell cycle entry are mutually exclusive, perhaps due to an energetic insufficiency preventing these programs from coexisting.

Consistent with a role for GPR3 as a constitutively active Gs-coupled GPCR, silencing GPR3 leads to hyperphosphorylation of CRTC2, a PKA-SIK axis substrate, suggestive of activation of CREB-dependent transcription^36^. However, we see no evidence of steady-state activation of CREB targets in cells lacking GPR3 (data not shown). Treatment of human islets cells lacking GPR3 with pan-SIK inhibitor prevents hyperphosphorylation of CRTC2, implicating SIKs as critical effectors downstream of GPR3. While our data support a GPR3-SIK2-CDKN1B pathway as central to regulating β cell quiescence, silencing GPR3 also reduced levels of CDKN1A which were not restored by inhibiting SIKs, pointing to the existence of additional effectors downstream of GPR3 that mediate CDKN1A status. However, silencing SIK2 in cells lacking GPR3 restored quiescence, and overexpression of SIK2 in the absence of TAg in human β cells promoted proliferation in vitro and in vivo, activation of SIK2 alone is necessary and sufficient for cell cycle entry. β cell knockout of SIK2 results in impaired glucose-stimulated insulin secretion via reduced phosphorylation of CDK5R1/p35 and inactivation of voltage-gated calcium channels, without a change in β cell mass^37^, suggesting compensation from SIK1 and SIK3 in this setting. Taken together, we propose that fluctuating SIK2 levels, dictated by ambient glucose concentrations, are a key element of the homeostatic process that fine-tunes the insulin secretory response to meet demand. Should hyperglycemic conditions persist, sustained increases in SIK2 levels lowers the threshold for cell cycle entry via a reduction in CDKN1B (Fig. 6h). During this mass expansion, SIK2 Tg mice increase their responsiveness to amino acids, consistent with the functional profile of fetal β cells^40^ and stem cell-derived β-like cells^42^. Our data indicate that a successful regenerative approach to increasing functional β cell mass will involve the pharmacologic management of both cell cycle entry and of maturation.

Chemical biology approaches have identified candidate small molecule activators of β cell proliferation, including harmine, a pan-kinase inhibitor that induces 1% of human β cells to enter the cell cycle^11^, by a mechanism that may involve as many as 14 kinases in the CMGC branch of the kinome^43^. Silencing harmine in cells increased proliferation by 1.8% but had no effect when added to cultures silenced for GPR3 in the context of TAg expression (20% vs 21% when harmine added, Supplementary Fig. 11). Harmine also reversibly inhibits monoamine oxidase A and has been evaluated as a mood-altering therapy in human subjects^44^. As several brain regions in GPR3 KO mice showed reduced levels of monoamine neurotransmitters such as serotonin and dopamine^45^, the connection between GPR3 and the regulation of monoamine oxidases and their substrates warrants further investigation. We contend that identification and validation of gene targets that establish and maintain β cell quiescence will require a genetic approach, which will, in turn, provide a comprehensive functional framework to design strategies that allow precise control over their proliferative and functional behavior.

Taken together, our work demonstrates the promise of functional genetic screens for dissecting therapeutically relevant state changes in primary human cells and demonstrates that GPR3 suppression of SIK2 activity is a key element of the network dedicated to repressing adult human β cell replication. As such, GPR3 and SIK2 represent targets for promoting the proliferation of human β cells to increase β cell mass for the treatment of insulin insufficiency. We anticipate that validation of additional candidates from the screen will provide additional cues about pathways that govern adult human β cell quiescence, and broader application of RNAi screening in primary human cells to inform regenerative approaches designed to elicit expansion of other mature, non-dividing cell populations for therapeutic advantage.

## Methods

### Animals

All procedures involving mice were approved by the Animal Care Committee of Sunnybrook Research Institute. SIK2 β cell transgenic mice were prepared by injection of a linearized SIK2-V5 tagged cDNA under the control of the mouse insulin promoter (MIP-SIK2-V5) into pseudopregnant C57Bl6 blastocysts. Two founder lines were positive for SIK2-V5 protein in isolated islets by Western blotting, and these were expanded for analysis (Founder 1: Gene Targeting Facility, University of Connecticut, CT, USA; Founder 2: the Center for Phenogenomics, Toronto, CA). Non-transgenic littermates were used as controls. For measurement of fasted and refed blood glucose levels, blood from males and females was analyzed using a OneTouch Ultra glucometer following a 16 h fast with water, then again after 1 h of refeeding with chow diet.

### Glucose/arginine tolerance test

Male and female mice were fasted for 16 h and injected IP with 2 g/kg body weight of 20% glucose (SIGMA G7021) in PBS or 1 g/kg body weight of 10% L-Arginine (SIGMA A8094) in PBS. For insulin measurements, blood (50 ul) was collected in hepatized capillary tubes (FisherBrand 22-260-950) at the indicated times. Samples were clarified by centrifugation at 15,000×*g* for 3 min and stored at −80 °C prior to the assay with the Ultra-Sensitive Mouse Insulin Kit (Crystal Chem 90080) according to the manufacturer’s protocol.

### Antibodies/reagents

Western Blot: Erk2 (Santa Cruz 1:1000), CDKN1A/p21 (Cell Signaling 1:1000), CDKN1B/p27 (Cell Signaling 1:1000), PTEN (Cell Signaling 1:1000), pAKT (Cell Signaling, 1:1,000), AKT (Cell Signaling 1:1,000), CDKN2C/p18 (Abcam 1:2000), PDX1 (Cell Signaling 1:1000), BclXL (Cell Signaling 1:1000), HSP90 (Santa Cruz 1:1000), V5 (Cell Signaling 1:1000), SKP2 (Cell Signaling 1:1000), CDKN1C/p57 (Cell Signaling 1:1000), CDKN2A/p16/INK4a (Cell Signaling 1:1000), CDKN2B/p15/INK4b (Abcam 1:1000) and SIK2 (Cell Signaling 1:1000). Immunofluorescence: insulin (Dako 1:1000), PDX1 (Abcam AB47383 1:2000), cleaved caspase 3 (Cell Signaling 1:100). The C-peptide antibody was from the Developmental Studies Hybridoma Bank, created by the NICHD of the NIH and maintained at The University of Iowa, Department of Biology, Iowa City, IA 52242. Antibodies for CRTC2 and pSer275 CRTC2 were described previously^38^. Click-it EdU Alexa Fluor 647 imaging kit (Life Technology) was used according to the manufacturer’s recommendations. Harmine was purchased from Cayman Chemicals. GNF4877 was a gift from J. Annes. Plasmids are described in Supplementary Data 5.

### Cell and islet culture

HEK293T-17 cells (ATCC) are grown in DMEM media supplemented with 10% FBS, 50 IU penicillin, and 50 µg/ml streptomycin. Human islet studies were approved by the Research Ethics Board of Sunnybrook Research Institute (study number 1540, entitled Human Pancreatic Beta Cell Regeneration). We used four sources of human pancreatic islets for assay development: the NIDDK-funded Integrated Islet Distribution Program (IIDP islets); University of Alberta, Edmonton, Canada, Clinical Islet Lab of J. Shapiro (Shapiro islets), Alberta Diabetes Institute Research Islet Lab, Canada, lab of P. MacDonald (ADI IsletCore islets), and Toronto University Health Network group, M. Cattral. Donor permission for research use was obtained by isolation centers. Supplementary Data 3 provides complete donor information and the experiments for which individual subjects were used. Male and female deceased donors were used, ranging in age from 17 to 78 years old and BMI from 18 to 44.4 kg/m^2^, none of which had a prior diagnosis of diabetes. When islet purity was under 85%, islets were picked manually and cultured for up to 14 days at 37 °C in a 5% CO_2_ atmosphere in non-tissue culture treated petri dishes in PIM(S) media supplemented with 5% human AB serum, glutamine/glutathione mixture and penicillin/streptomycin (all reagents from Prodo Laboratories Inc.). The medium was changed every 2–3 days.

### GSIS

Intact human islets (50 islets per replicate) or re-aggregated (5000 cells/well in V-bottom 96-well plate) human islets were equilibrated in Krebs ringer buffer (KRB) containing 2.8 mM glucose for 30 min, then incubated in 2.8 mM glucose in KRB for 1 h prior to stimulation with 16.7 mM glucose in KRB for 1 h, followed by 45 mM KCl for 1 h. KRB supernatants from 2.8 mM, 16.7 mM glucose, and 45 mM KCl treatments were collected, and insulin amount determined using a human insulin HTRF assay (Cisbio). Cells infected with lentivirus were incubated for 6 days prior to the GSIS assay. All conditions were done in triplicate or quadruplicate.

### Islet dissociation and seeding

Islets were washed in PBS and dissociated with Accutase (1 ml/1000 IEQ) for 5–7 min at 37 °C and triturated every 60 s. Dissociated islet cells were seeded at a density of 15,000 cells/well in a 384-well plate for fluorescence or 60,000 cells/well in a 96-well plate to generate protein extracts. Islets were always seeded on a PDL-coated plate to facilitate attachment of dissociated cells^12^.

### Lentiviral shRNA

Plasmids were from the MISSION Human shRNA library (Millipore SIGMA). The pLKO.1eGFP control vectors (EV, NT, CDKN2C/p18, CDKN1A/p21) and pLenti6.3-SV40T Antigen-V5 (TAg), Lentivirus purification was achieved by ultracentrifugation for 2 h at 96,000×*g* at 4 °C, and the viral pellet was resuspended in PBS and frozen at −80 °C for future use^35^.

### HTS plasmid purification

GPCR genes were identified using data from the IUPHAR GPCR list and UNIPROT data for known 7 transmembrane-spanning receptors (accessed November 2016). Known pseudogenes were excluded. The resulting list of 437 genes consisted of 397 GPCRs and 40 GPCR-related and associated proteins. The shRNAs for 7 GPCRs and 2 GPCR-related proteins were unavailable in the Mission TRC library resulting in 98% coverage of the selected GPCRome and a total of 2342 shRNAs. In total, 1–26 shRNAs (average = 5) were available per gene (Supplementary Data 2). Frozen glycerol stocks from the GPCR shRNA gene set were scraped, inoculated into 4 ml of TB medium containing 50 μg/ml ampicillin in a 24-well deep-well culture plate, and grown for 18 h at 37 °C with 250 rpm shaking. Bacteria were pelleted by centrifugation at 1000×*g* for 10 min. Plasmids were isolated using 96 well NucleoSpin transfection-grade DNA kits (Machery-Nagel) measuring absorbance at 260 nm and 280 nm and verified by agarose gel electrophoresis. DNA was diluted to 10 ng/ml in 5 mM Tris/HCl (pH 8.5) using a Mantis Liquid Handler (Formulatrix), arrayed, and stored in a robotic −20 °C storage system (Hamilton Verso).

### HTS lentiviral packaging

Viral packaging (pCMV8.74, 45 ng/well), envelope (pMD2G, 6 ng/well) and shRNA-containing transfer (pLKO.1-shRNA, 50 ng/well) plasmids were combined in 20 μl/well OptiMEM (Life Technologies) containing 0.2 μg/well linear polyethyleneimine 25,000 (PEI) transfection reagent in a 96-well tissue culture plate. Following incubation for 15 min at room temperature, 10^5^ HEK293T-17 cells in 100 μl DMEM containing 10% FBS and 50 IU/ml penicillin/50 μg/ml streptomycin were added to the DNA mixture, mixed by pipetting, and incubated at 37 °C 5% CO_2_ for 72 h. HEK293T-17 cells were maintained below 90% confluence for no more than 20 passages.

### Lentiviral purification

Cell supernatants were vacuum filtered (0.2 μm, 96-well—Agilent Technologies) and viral particles were mixed with polyethylene glycol 8000 (SIGMA) to a final concentration of 5–6% and incubated at 4 °C for 5 h. Lentivirus was pelleted by centrifugation at 2500×*g* for 1 h and resuspended in a 30 μl PIM(S) medium. Purified lentivirus was arrayed in black 384-well poly-D-lysine-coated imaging plates (Greiner) and stored at −80 °C until use^12^.

### shRNA-resistant cDNA rescue

The open reading frame of GPR3 cDNA (Genscript) and SIK2^36^ was cloned into a modified version of pLenti6/V5-DEST vector (Invitrogen) in which we deleted 300 nucleotides of the CMV promoter distal to the transcription start site were removed to attenuate its potency as per^46^. The resulting expression plasmid, pLenti6-delta4/V5-DEST provides more physiological expression levels appropriate for genetic rescue experiments. shRNA-resistant constructs were generated by QuickChange Lightning Site-Directed Mutagenesis Kit (Agilent). PCR oligo sequences and mutagenesis primer sequences are listed in Supplementary Data 4.

### Islet cell infection

Dissociated islet cells were infected at the time of seeding by adding the cell suspension to multi-well plates (96 or 384-well) in which purified lentivirus had been arrayed. For high-potency concentrated lentivirus prepared by ultracentrifugation^12^, islet infection in 384 well plates was performed using 0.5% of the yield from a 10 cm dish for all viruses. For 96 well plates, 5% of the yield was used. For GPCR screen shRNAs, islets in 384-well plates were infected with 100% of yield from a 96 well dish of PEG-purified lentivirus. For epistasis experiments, one shRNA virus (CDKN2C-sh, CDKN1A-sh, or CDKN1B-sh) was added together with lenti-TAg at the time of seeding; 24 h later cells were rinsed twice with PIMS medium, and the second virus (GPR3sh) was added. Harmine (10 uM) and GNF4877 (2 μM) were added to wells 3d and 7d after seeding prior to imaging on d10.

### Immunofluorescence

EdU was added to the medium at 10 μM on 3 and 7 days after seeding. Following 10 days of lentiviral infection, dissociated islet cells were fixed by adding 3.7% paraformaldehyde containing Hoechst (5 μg/ml) for 15 min at 37 °C and quenched with an equal volume of 0.75% glycine in PBS for 5 min. Cells were permeabilized with 0.1% Triton-X-100 in PBS for 10 min. Click-IT EdU detection was performed according to the manufacturer’s protocol, prior to blocking in 3% BSA in PBS overnight. Antibodies in 3% BSA were incubated overnight at 4 °C (primary) or for 1 h at RT (secondary). Each step was followed by two gentle washes in PBS using a BioTek 405 select multiwell plate washer.

### Imaging and data analysis

Fluorescent images were captured using a Perkin-Elmer Opera Phenix automated confocal multiwell plate microscope fitted with a 40X high NA water lens. 49 fields per well were acquired in each fluorescence emission channel: blue 405 (Hoechst), green 488 (GFP), red 594 (C-peptide/Insulin/PDX1), and far-red 647 (EdU). Columbus high-content imaging analysis software was used to generate an algorithm to identify the intensity of each stain in regions of interest in an object (cells) and to determine the % of nuclear EdU+ (proliferating) and C-peptide+ (insulin-positive) β cells that were GFP+ (infected with lenti-shRNA virus).

### RT-PCR

In total, 400,000 cells from dissociated islets were infected with NT or GPR3 shRNA lentivirus in a poly-D-lysine-coated 24 well plate. Six days post-infection, cells were collected and lysed with TRIZOL (manufacturer) and total RNA was isolated according to the manufacturer’s protocol. Any contaminating DNA was removed from the sample using the DNA-free DNase Treatment and Removal Kit (Thermo Fisher Scientific) according to the manufacturer’s protocol. Total RNA was transcribed into cDNA using random primers using the Superscript III First-Strand Synthesis kit (Thermo Fisher Scientific) and amplified with PDX1 and GPR3-directed oligos described in Supplementary Data 4 and Supplementary Fig. 4. PCR cycling conditions for PDX1 were: 95 °C for 5 min, 95 °C for 30 s, 54 °C for 30 s, extension at 72 °C for 30 s (30 cycles), followed by a final extension at 72 °C for 10 min. PCR cycling conditions for GPR3 were: 95 °C for 5 min, 95 °C for 30 s, 68 °C for 30 s, extension at 72 °C for 30 s (3 cycles), 95 °C for 30 s, 65 °C for 30 s, extension at 72 °C for 30 s (3 cycles), 95 °C for 30 s, 62 °C for 30 s, extension at 72 °C for 30 s (3 cycles), 95 °C for 30 s, 60 °C for 30 s, extension at 72 °C for 30 s (25 cycles), followed by a final extension at 72 °C for 10 min.

### SIK inhibitor treatment

Dissociated islets were plated on either 384-well plates (for imaging) or 96-well plates (for Western blots) and infected with the indicated lentivirus. Twenty-four hours after infection, cells were treated at a final concentration of 2 μM HG-9-91-01 (APExBIO) for the duration of the experiment. The fresh drug was added daily.

### Western blotting

Six days after lentiviral infection, dissociated human islet cells were washed twice with ice-cold PBS prior to lysis in 2× Laemmli buffer. Sample proteins were separated by SDS-PAGE electrophoresis and transferred to a low-fluorescence PVDF membrane (Millipore). Membranes were blocked for 1 h in 3% milk in TBST prior to incubation with the indicated primary antibodies in 3% BSA overnight at 4 °C. IRDye secondary antibodies (Licor) were diluted 1:30,000 (IR680) or 1:18000 (IR800) in 3% milk in PBS containing 0.01% SDS and 0.04% TritonX-100 and incubated with membranes for 1 h at room temperature. A Licor Odyssey Clx was used for signal detection. All Western blots are representative of at least *n* = 3 experiments.

### β cell proliferation in vivo

Pancreases from 12 to 16 weeks old female WT (*n* = 2), SIK2 Tg founder 1 (*n* = 3), and founder 2 (*n* = 3) mice were isolated and fixed O/N in 4% paraformaldehyde/PBS, then rinsed with 70% ethanol prior to paraffin embedding at the Histology Core at Sunnybrook Research Institute (SRI). Blocks were then sectioned and stained for Hoechst (blue), Insulin (Green), and Ki67 (Red) at the Histology Core at the University Health Network. The percentage of Ki67+ insulin+ cells in islets was determined from three stained sections per mouse ~150 μm apart. The stained sections were imaged using the Perkin-Elmer Opera Phenix with a 40X high NA water lens.

### β cell area

Pancreases were sent to Histology Core at SRI for H&E stain and paraffin embedding. Pancreatic sections were taken 150 μm apart, and deparaffinized prior to antigen retrieval in 0.1 M sodium citrate pH6 and staining with guinea pig anti-insulin antibody (Dako A0564, 1:250) overnight at 4 degrees in the dark and visualized with Anti-Guinea Pig biotin-conjugated antibody (Vectastain BA7000, 1:300). Slides were imaged at the Advanced Optical Microscopy Facility, Toronto, and images analyzed using Aperio ImageScope.

### Statistics and reproducibility

Statistical analyses were performed using GraphPad Prism® 8 or Excel Software. Statistical significance was determined using Student’s *t*-test when comparing two means; one-way ANOVA and Tukey post-tests were applied for comparison between different groups. *p*-values of < 0.05 were taken to indicate statistical significance (ns or no asterisk = not significant, *p* ≥ 0.05, *p ≤ 0.05, **p ≤ 0.01, ***p ≤ 0.001, ****p ≤ 0.0001). Unless otherwise indicated, error bars represent ± standard error of the mean across three independent donors/experiments.

### Reporting summary

Further information on research design is available in the Nature Research Reporting Summary linked to this article.

## Supplementary information

Supplementary Information

Supplementary Data 1

Supplementary Data 2

Supplementary Data 3

Supplementary Data 4

Supplementary Data 5

Description of Supplementary Files

Reporting Summary

## Data Availability

The authors confirm that all relevant data are included in the paper and/or its supplementary information files. Original scans for Western blots are provided in Supplementary Fig. 12 and source data files are provided in Supplementary Data. pLenti6.3-delta4-GPR3-V5 has been deposited at Addgene as #172601.
