# Supplementary material for: Silencing the G-protein coupled receptor 3-salt inducible kinase 2 pathway promotes human β cell proliferation
Source: Commun Biol. 2021 Jul 23;4:907. doi: 10.1038/s42003-021-02433-2 (PMC8302759; doi:10.1038/s42003-021-02433-2)
Supplement: Supplementary file 1 — Supplementary Information [file 42003_2021_2433_MOESM1_ESM.pdf]

Supplementary Figure 1. Beta cell identity is not lost over 10-day screening assay timecourse.

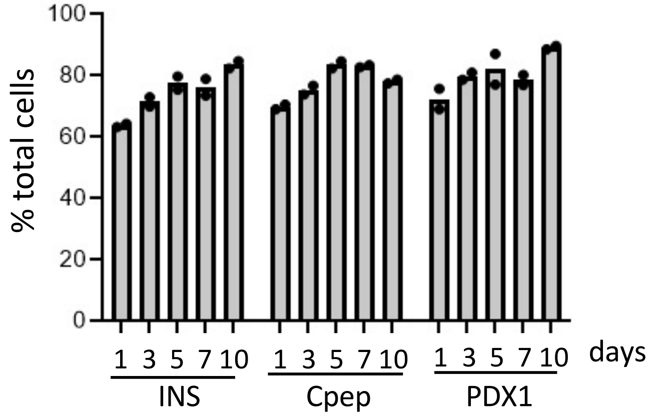

Barplot showing percentage of beta cells over screening assay time course, indicated by insulin (INS), C-peptide (Cpep) and PDX1 staining. n=2.

Supplementary Figure 2. Beta cell survival is not compromised during 10-day assay timecourse.

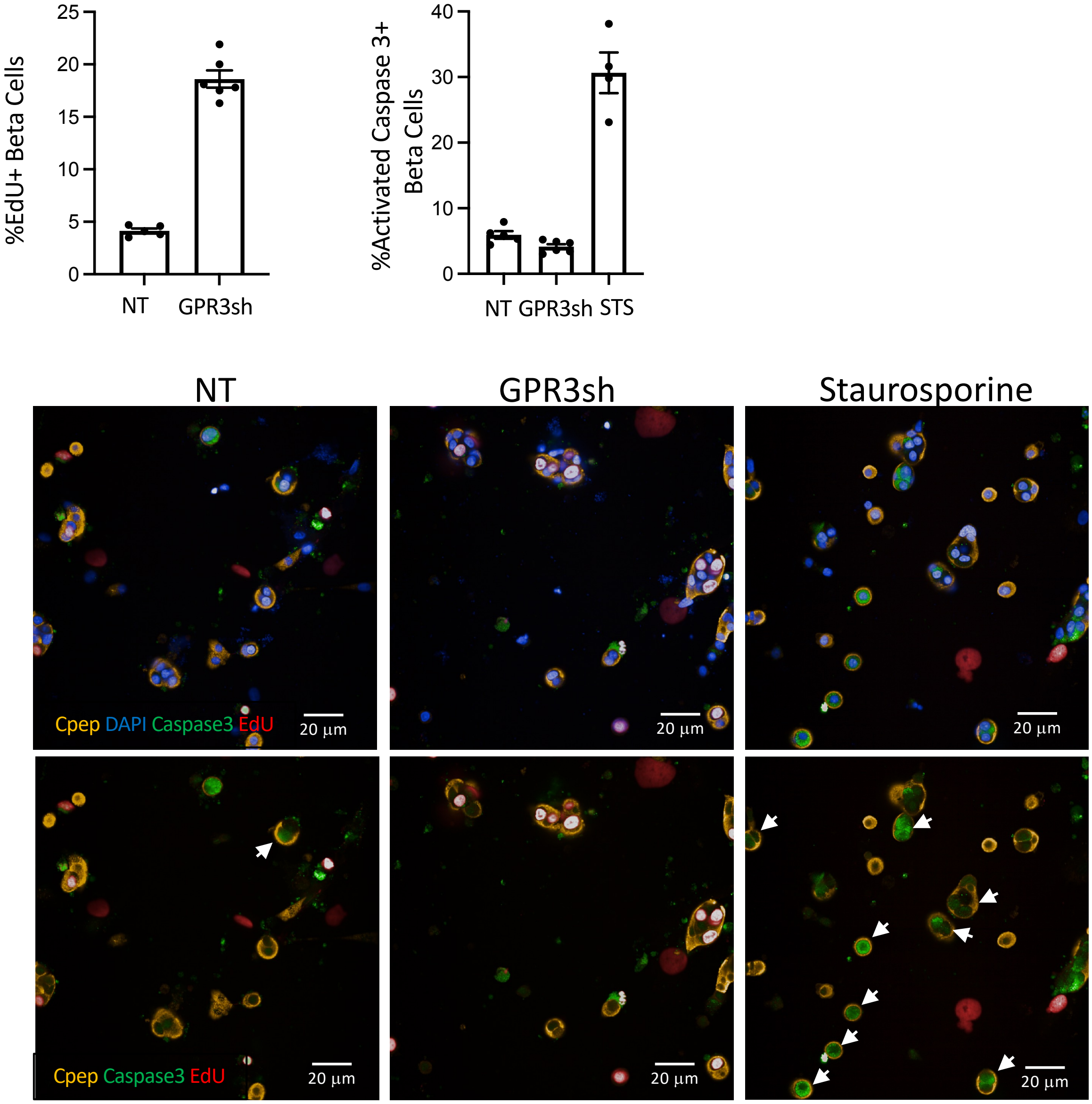

Top: % EdU+/C-peptide+ cells (left) and % activated-caspase3/C-peptide+ cells (right) in human beta cells silenced for GPR3 compared to non-targeting control (NT). Effect of the apoptotic stimulus staurosporine (40 mM) is shown (16 hr treatment). Barplot depicted is a representative plot from 3 independent donors. Error bars represent the standard error of the mean from 3 technical replicates per donor.

Bottom: Immunofluorescence micrographs of human beta cells silenced for GPR3 compared to non-targeting control (NT) stained with C-peptide (yellow), EdU (red), activated caspase-3 (green), and DAPI (blue). Effect of 40 mM staurosporine (STS) is shown. NOTE: activated caspase-3+ cells are not EdU+. Total number of cells counted = 2000.

Supplementary Figure 3. Glucose stimulated insulin secretion assay for islets from donors used for GPCRome screen.

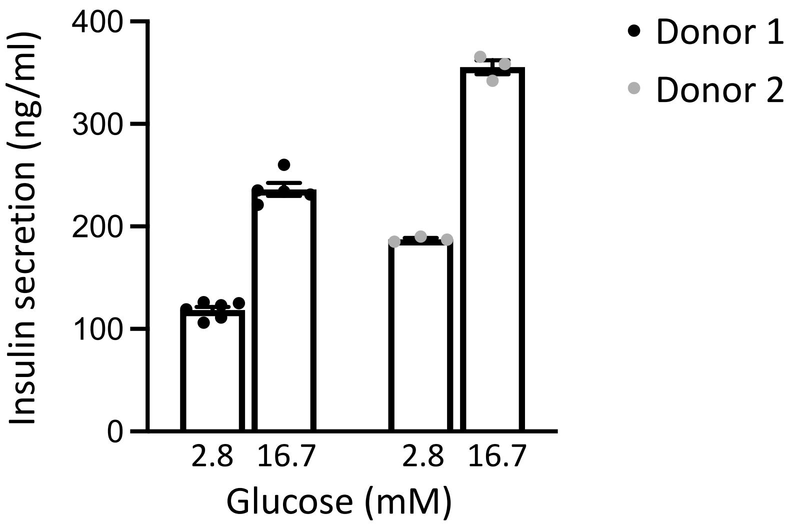

Static GSIS data for islets from two donors that were pooled and used for GPCR screen. Treatment with low (2.8 mM) and high (16.7 mM) glucose shown. Error bars represent the standard error of the mean for 6 technical replicates (donor 1) and 3 technical replicates (donor 2).

Supplementary Figure 4. RT-PCR analysis of total RNA from human islet cells silenced for GPR3 compared to non-targeting (NT) control shRNA. Positions of RT-PCR primers that flank introns are shown.

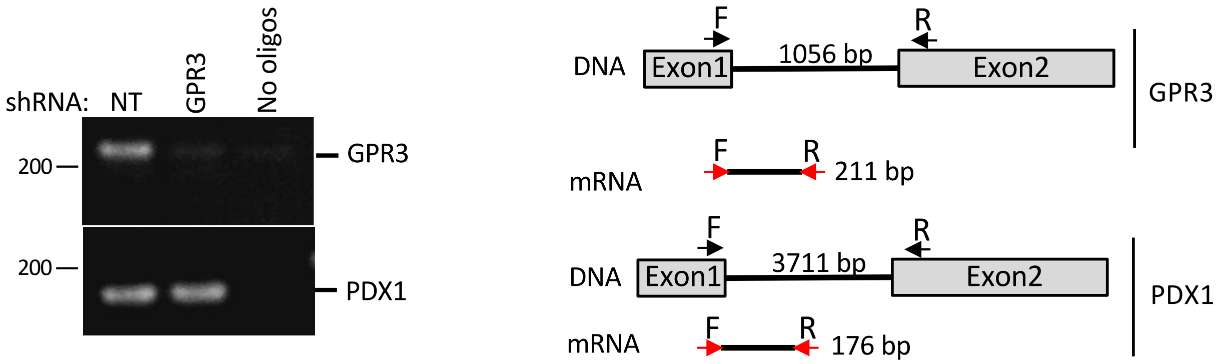

(left) RT-PCR data showing loss of GPR3 mRNA in cells silenced for GPR3. PDX1 internal control shown. (right) Schematic showing RT-PCR primers spanning exons.

Supplementary Figure 5. Proliferation of C-peptide+ islet cells in the presence and absence of co-infected TAg.

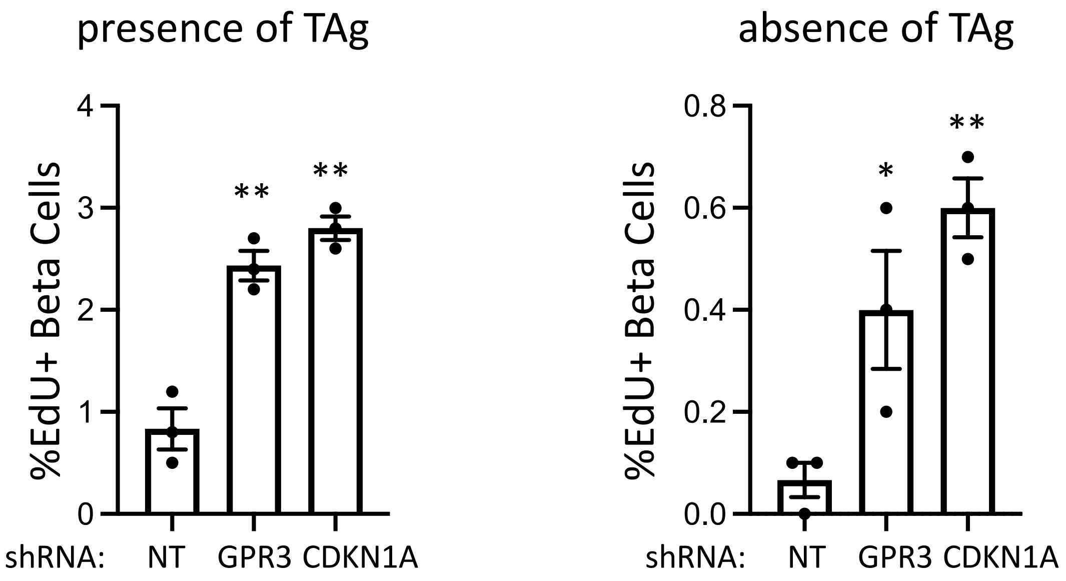

Beta cell proliferation in dissociated human islet cultures following introduction of non-targeting (NT), GPR3 or CDKN1A shRNA in the presence (left) or absence (right) of TAg. Barplots shown are representative plots from 3 independent donors. Error bars represent the standard error of the mean from 3 technical replicates per donor.

Supplementary Figure 6. Proliferation in C-peptide+ cells following silencing of GPR3 in the presence of co-expressed SIK1 or SIK3 shRNAs.

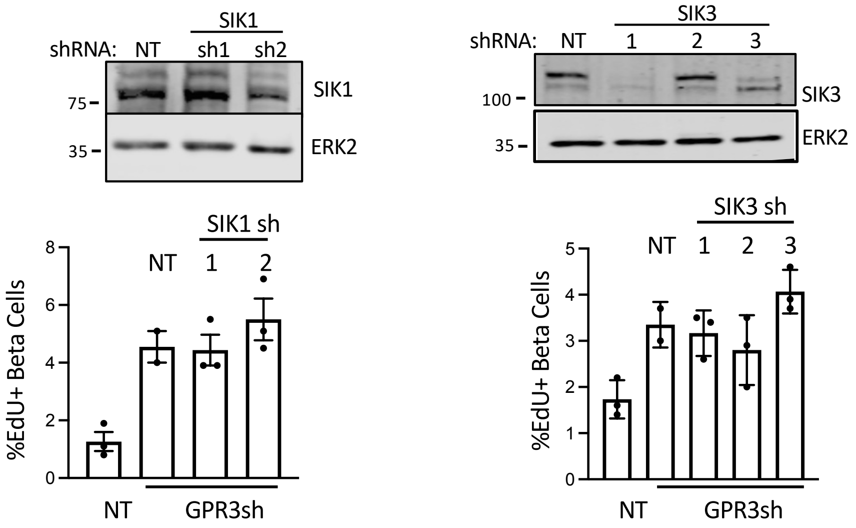

Silencing of SIK1 and SIK3 does not restore quiescence in cells silenced for GPR3. Barplots showing % proliferation with indicated combination of shRNAs and Western blots showing degree of silencing of SIK1 and SIK3 with indicated shRNAs is shown. Western blots and barplots shown are representative from 3 independent donors. Error bars represent the standard error of the mean from 3 technical replicates per donor. MW markers are in kDa.

Supplementary Figure 7. SIK2-V5 protein levels in SIK2 Transgenic founder 1 and SIK2 Transgenic founder 2.

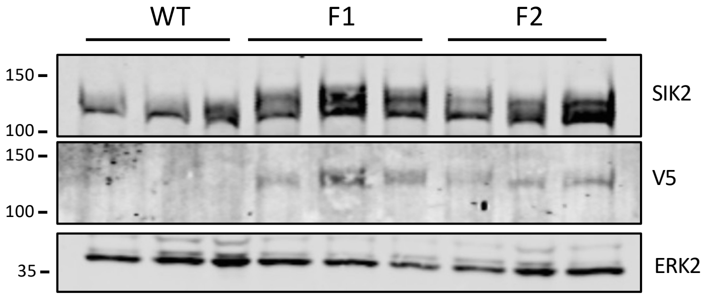

Western blots showing levels of SIK2 in WT, SIK2 Tg founder 1, and SIK2 Tg founder 2. Blot for V5 tag on transgene and ERK2 loading control shown. MW markers are in kDa.

Supplementary Figure 8. Glucose tolerance tests in female and male WT and SIK2 Tg mice.

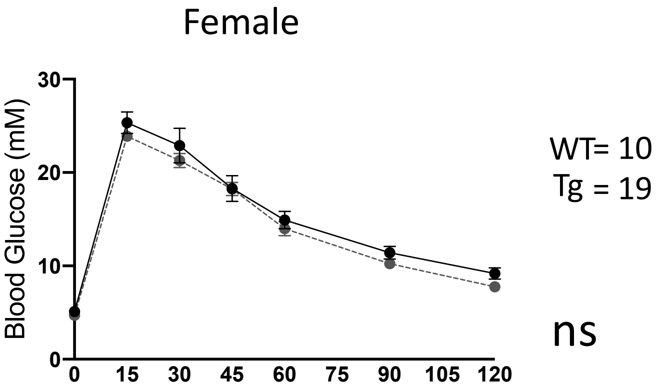

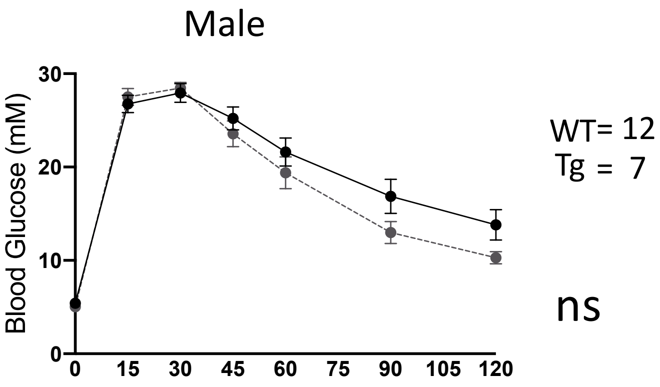

Glucose tolerance tests in female (left) and male (right) WT or SIK2 Tg 20-week-old mice. Error bars represent the standard error of the mean for 11 (WT male), 8 (Tg male), 12 (WT female), and 19 (Tg female) biological replicates.

Supplementary Figure 9. PDX1 protein levels are unchanged in islets from human subjects with increasing BMI.

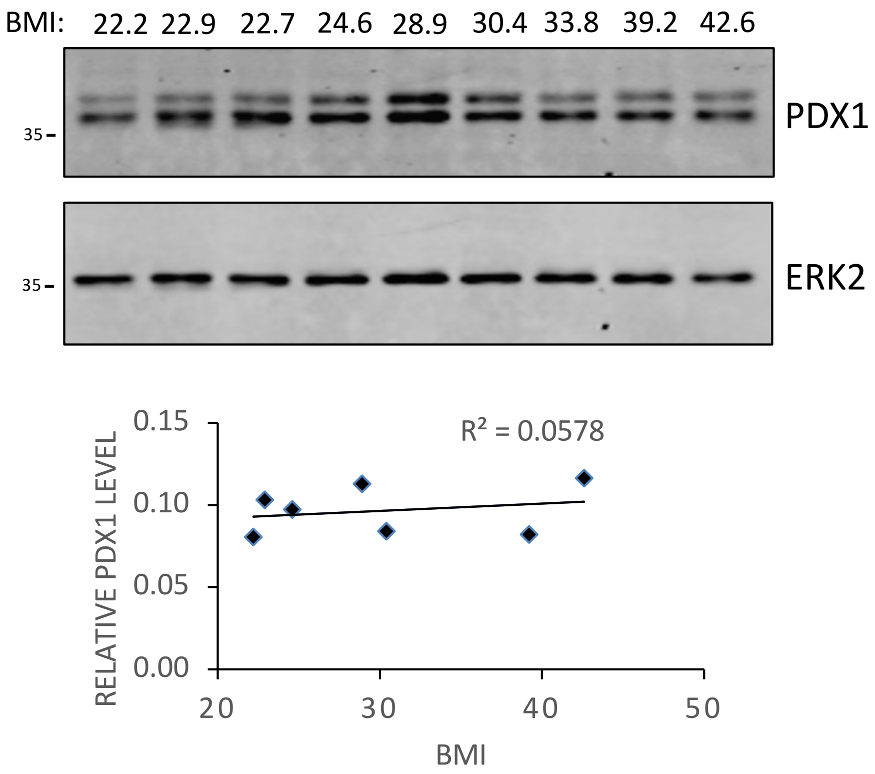

PDX1 blot for human subjects of increasing BMI. Loading control ERK2 is shown. MW markers are in kDa. Quantification and R^2 value shown.

Supplementary Figure 10. Proliferation in human alpha and delta cells following silencing of GPR3 in the presence of TAg.

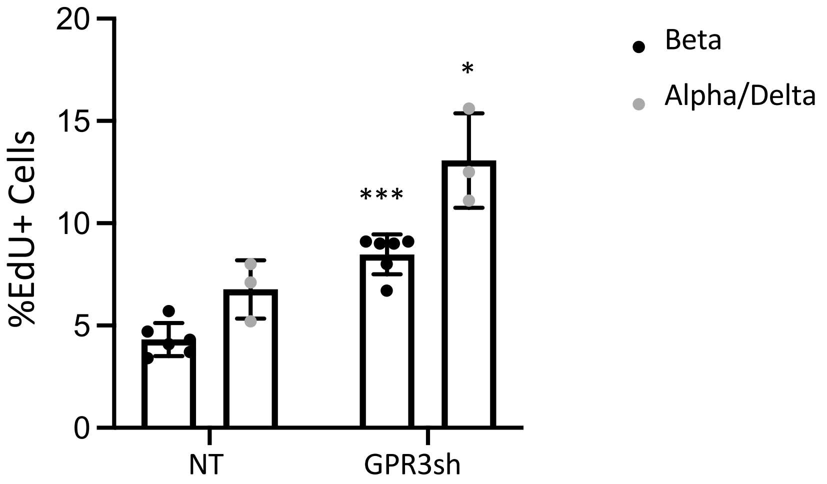

Beta cell or alpha/delta cell proliferation in dissociated human islet cultures following introduction of non-targeting (NT) or GPR3 shRNA in the presence of TAg. Barplot shown is representative of 3 independent donors. Error bars represent the standard error of the mean from 3 technical replicates per donor.

Supplementary Figure 11. Treatment of human islet cells with harmine does not increase proliferation in cells expressing TAg and lacking GPR3.

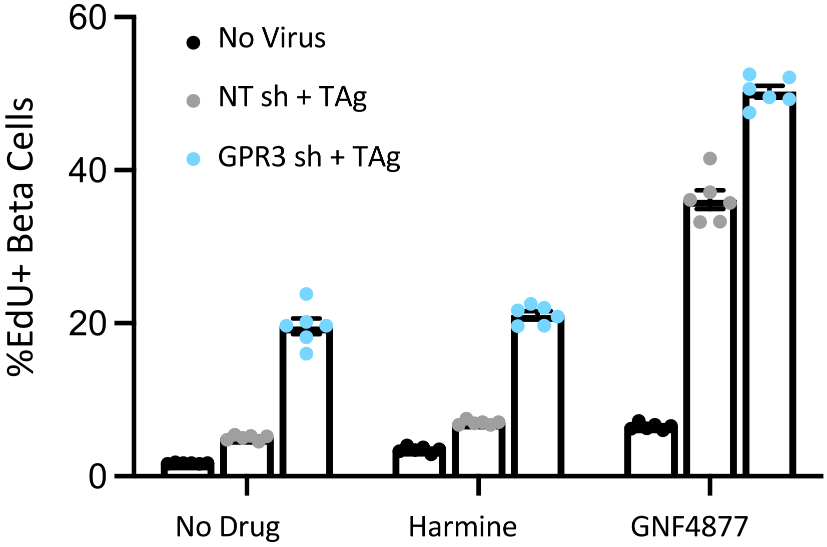

Barplot showing proliferation in human beta cells following 5d treatment with pan-kinase inhibitors harmine (10 uM) or GNF4877 (2 um) alone (black bars), in combination with SV40 TAg (white bars), or in combination with SV40 TAg and GPR3 silencing (grey bars). Barplot shown is a representative plot from 3 independent donors. Error bars represent the standard error of the mean from 6 technical replicates per donor.

Supplementary Figure 12. Uncut Western blots .

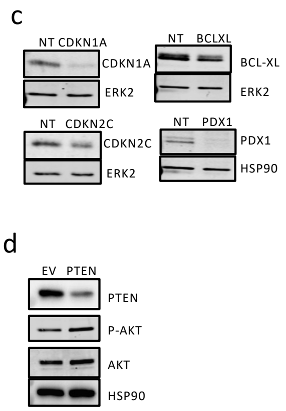

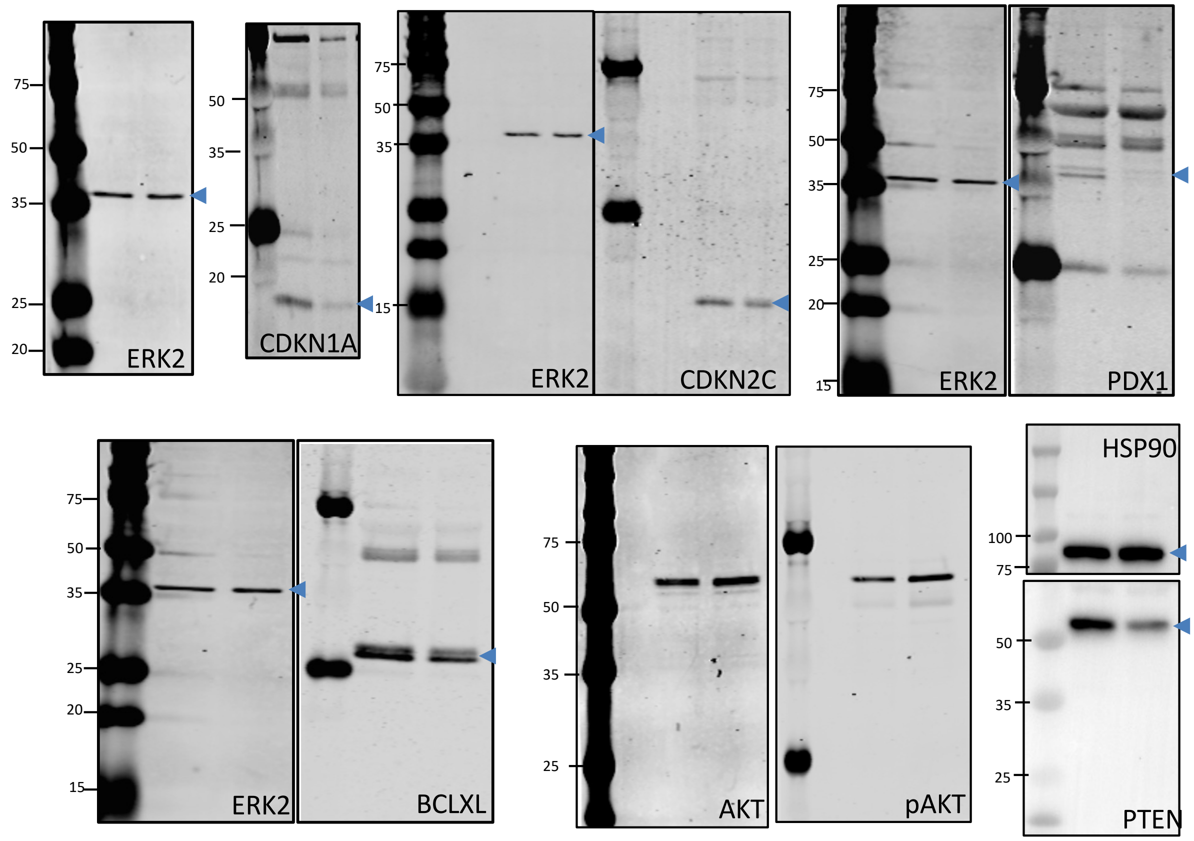

Figure 1 c. Representative Western blots showing knockdown efficiency of positive controls following infection with lentiviral-shRNAs against CDKN2C/p18, CDKN1A/p21, BCL-XL, and PDX1. d. Effect of silencing PTEN on downstream effector phospho-AKT, with total AKT and HSP90 control is shown. MW markers are in kDa.

Supplementary Figure 12. Uncut Western blots .

Uncut Western blots for Figure 2e.

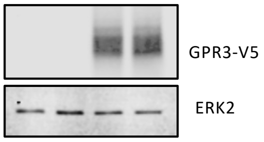

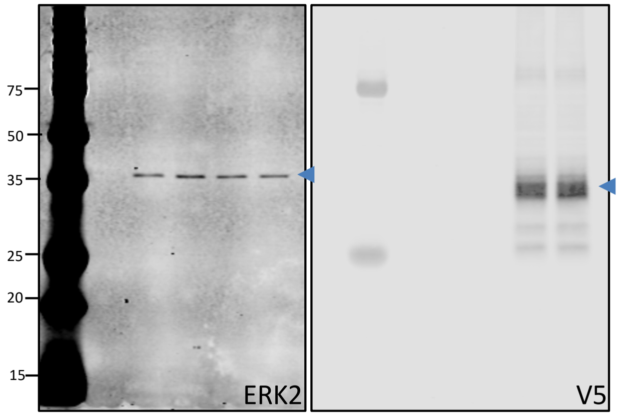

Western blots showing expression of V5-tagged GPR3 . MW markers are in kDa.

Supplementary Figure 12. Uncut Western blots .

Uncut Western blots for Figure 3a.

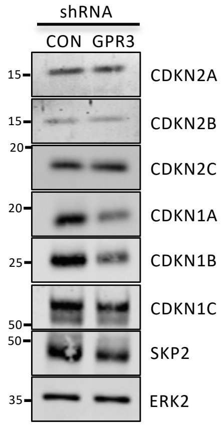

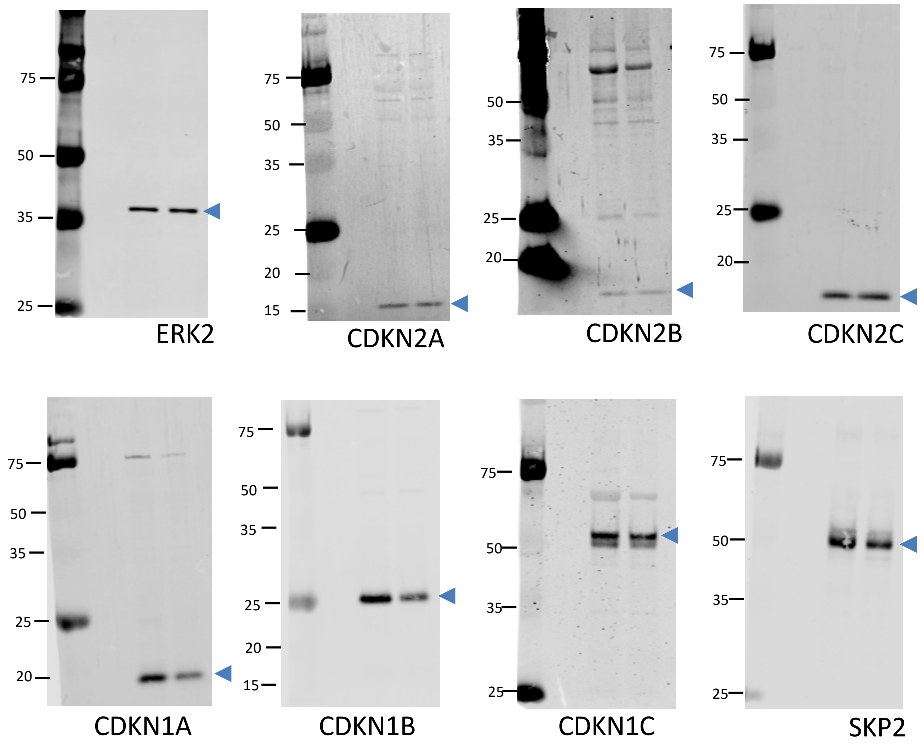

Representative Western blot analysis of INK and CIP/KIP family CKI levels in NT control and GPR3-silenced human islet cells. MW markers are in kDa.

Supplementary Figure 12. Uncut Western blots .

Uncut Western blots for Figure 3c-e.

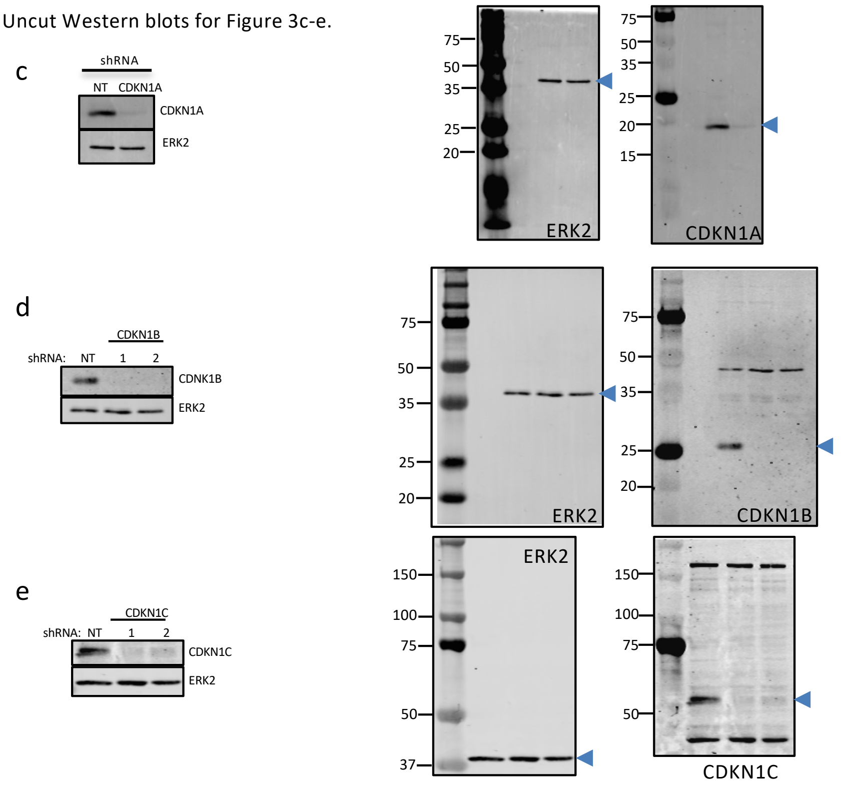

c. Western blot of CDKN1A following silencing of CDKN1A. d. Western blot of CDKN1B following silencing of CDKN1B. e. Western blot of CDKN1C following silencing of CDKN1C. MW markers are in kDa.

Supplementary Figure 12. Uncut Western blots .

Uncut Western blots for Figure 4a-b.

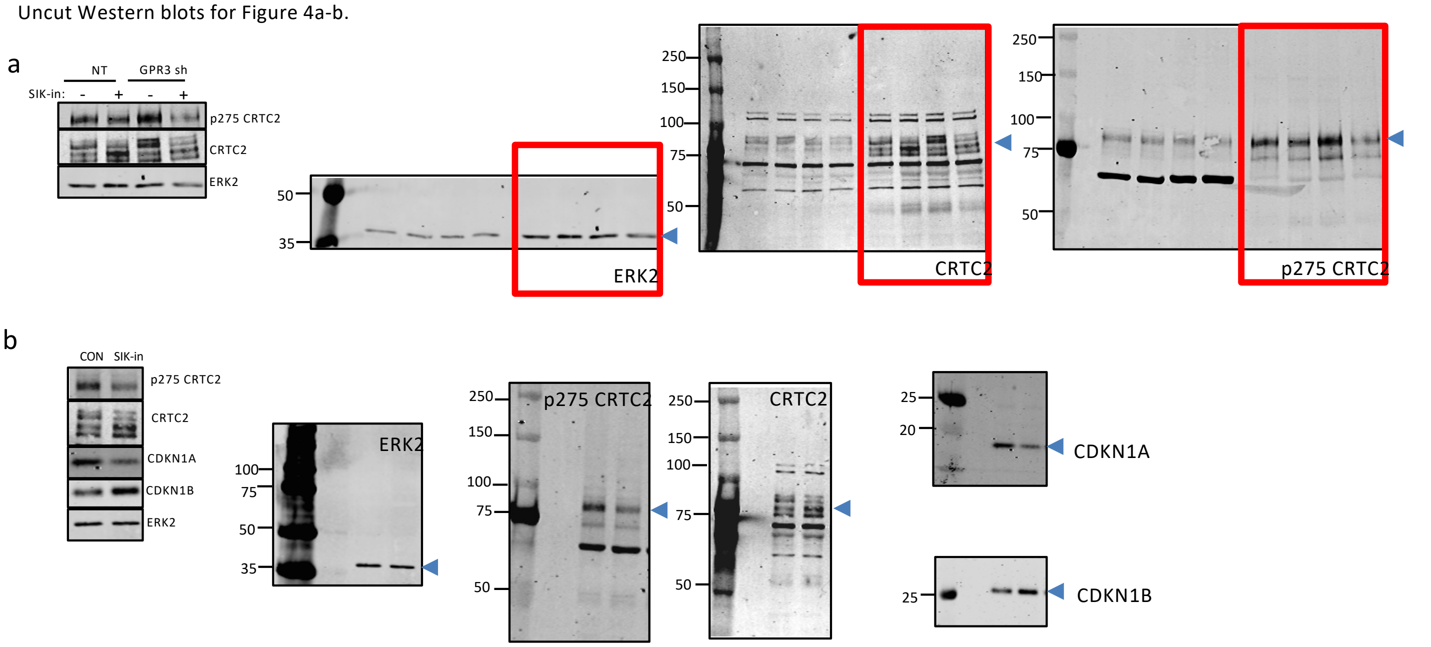

a. Western blot showing increase in phosphorylation status of SIK2 target protein CRTC2. Effect of pan-SIK inhibitor (SIK-in) is shown. b. Western blot showing effect of SIK-in on levels of CDKN1A and CDKN2B in human islet cells. MW markers are in kDa.

Supplementary Figure 12. Uncut Western blots .

Uncut Western blots for Figure 4d,f.

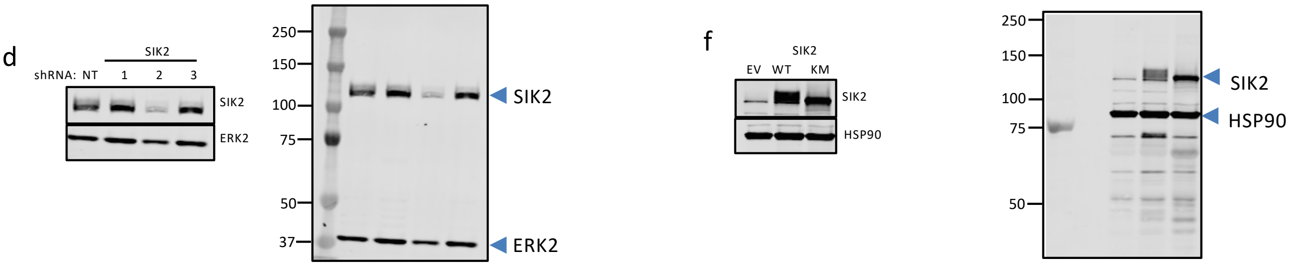

d. Western blots showing silencing of SIK2 with shRNA2. f. Western blot showing levels of SIK2 wild type (WT) and kinase dead (KM mutant) proteins. MW markers are in kDa.

Supplementary Figure 12. Uncut Western blots .

Uncut Western blots for Figure 4i.

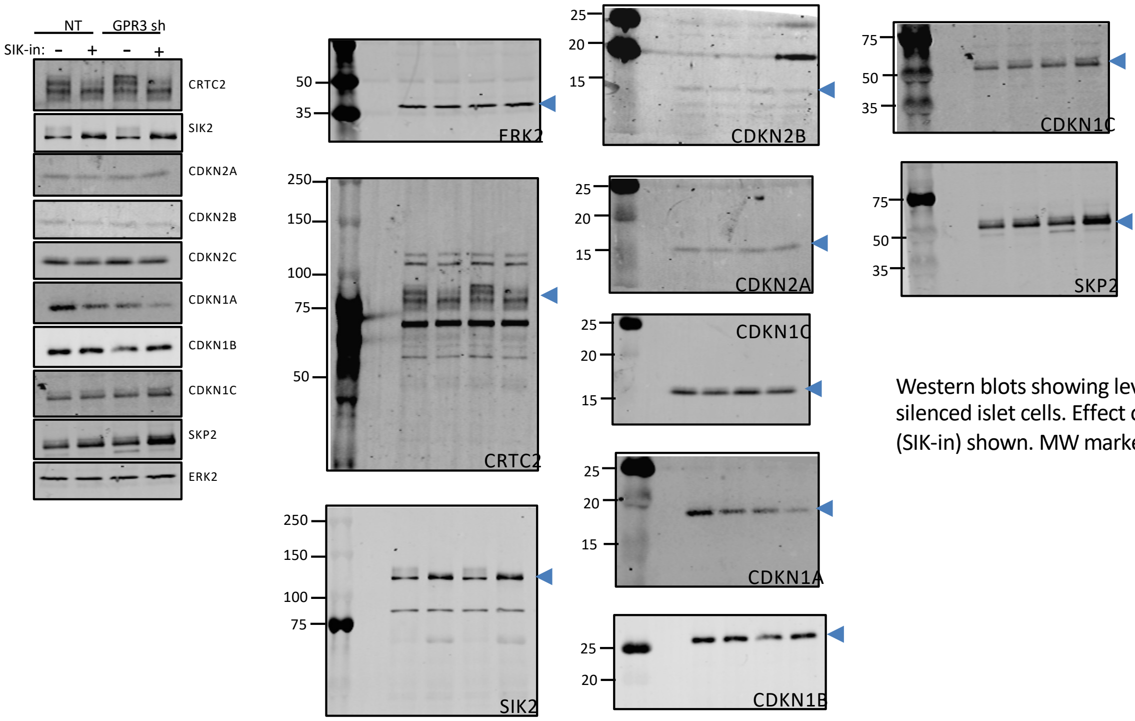

Western blots showing levels of CIP/KIP proteins in GPR3 silenced islet cells. Effect of pan-SIK inhibitor HG-9-91-01 (SIK-in) shown. MW markers are in kDa.

Supplementary Figure 12. Uncut Western blots .

Uncut Western blots for Figure 4j.

Western blots showing CDKN1B levels in islet cells following GPR3 silencing or overexpression of SIK2 WT or kinase dead mutant (KM). MW markers are in kDa.

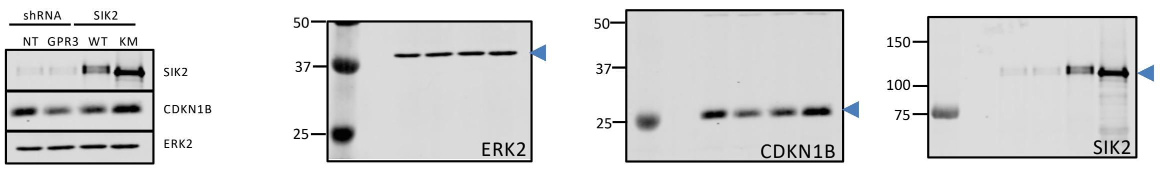

Supplementary Figure 12. Uncut Western blots .

Uncut Western blots for Figure 5a.

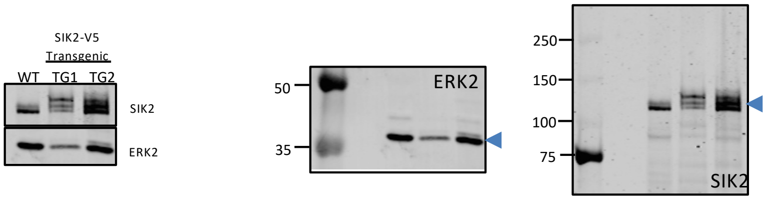

Western blot showing levels of endogenous and SIK2-V5 protein in control and MIP-SIK2 transgenic (SIK2-V5-TG) animals with ERK2 loading control. MW markers are in kDa.

Supplementary Figure 12. Uncut Western blots .

Uncut Western blots for Figure 6 a,c.

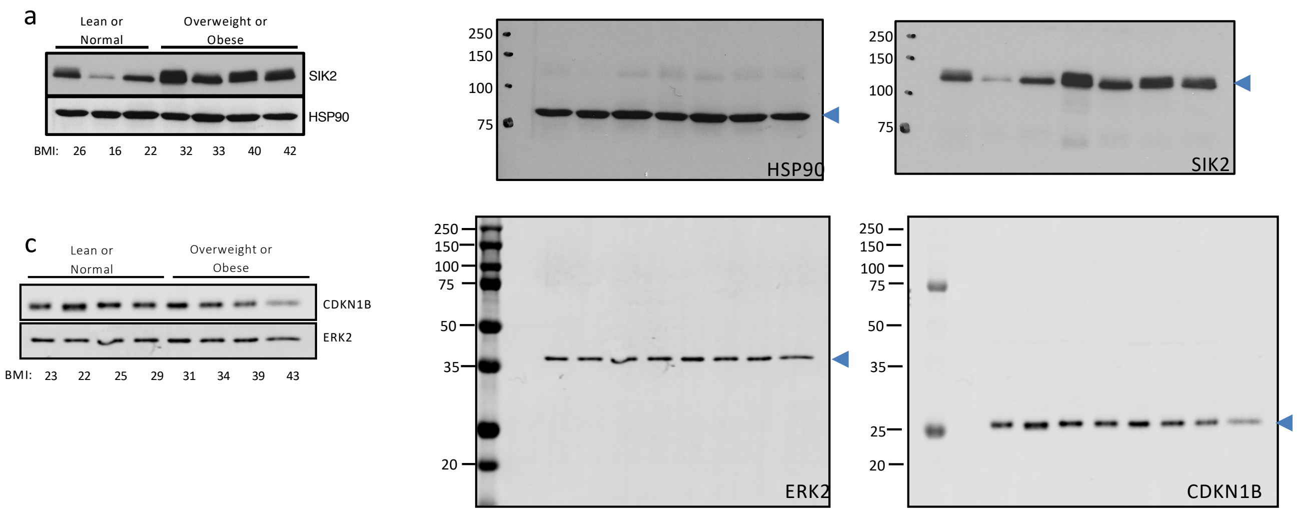

a. Western blots showing levels of SIK2 protein and HSP90 loading control in isolated human islets from non-diabetic human subjects of increasing BMI. c. Western blots showing levels of CDKN1B protein and ERK2 loading control in isolated human islets from non-diabetic human subjects of increasing BMI. MW markers are in kDa.

Supplementary Figure 12. Uncut Western blots .

Uncut Western blots for Figure 6e.

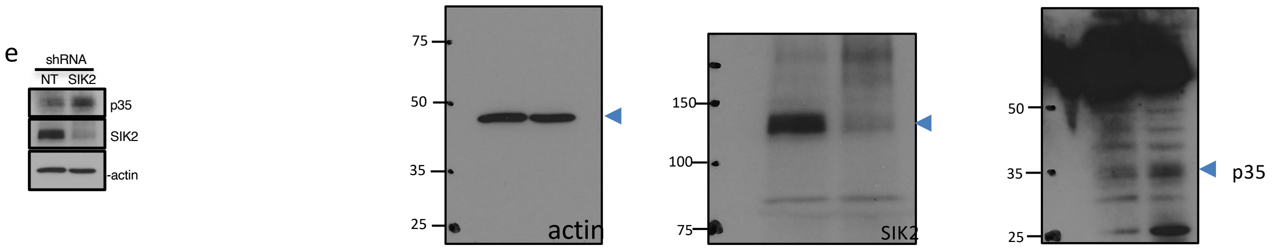

e. Western blot showing levels of SIK2 and its substrate CDK5R1/p35 following silencing of SIK2 compared to NT control. MW markers are in kDa.

Supplementary Figure 12. Uncut agarose gels .

Uncut Agarose gel for Supplementary Figure 4.

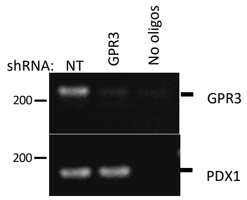

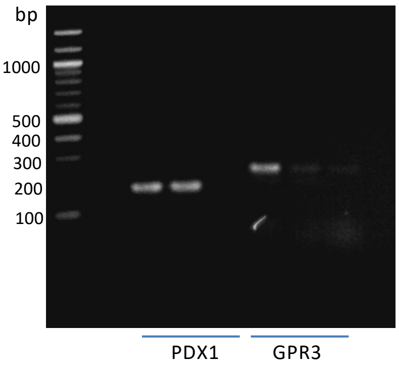

Uncut agarose gel showing RT-PCR data and loss of GPR3 mRNA in cells silenced for GPR3. PDX1 internal control shown. MW markers are in bp.

Supplementary Figure 12. Uncut Western blots .

Uncut Western blots for Supplementary Figure 6

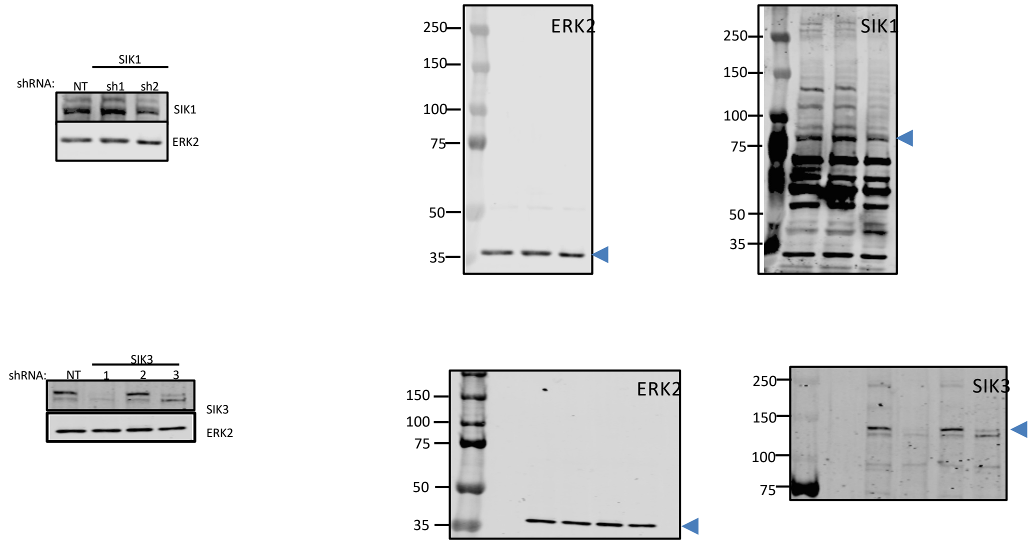

Western blots showing degree of silencing of SIK1 and SIK3 with indicated shRNAs. MW markers are in kDa.

Supplementary Figure 12. Uncut Western blots .

Uncut Western blots for Supplementary Figure 7.

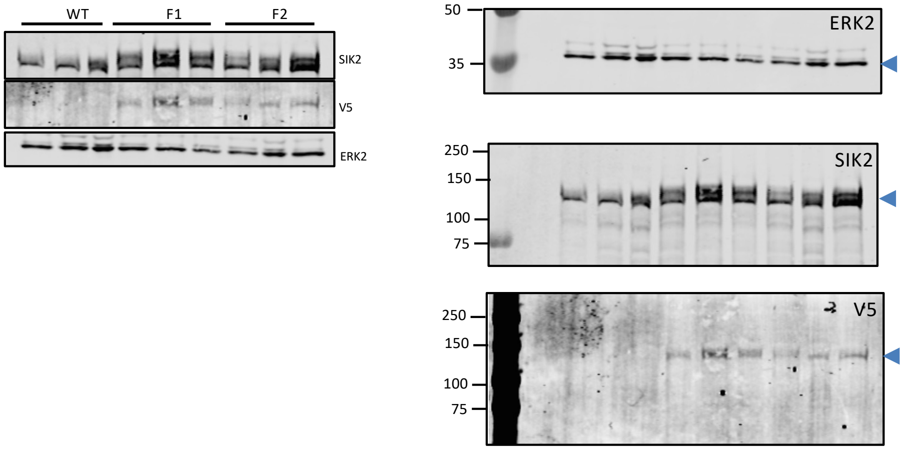

Western blots showing levels of SIK2 in WT, SIK2 Tg founder 1, and SIK2 Tg founder 2. Blot for V5 tag on transgene and ERK2 loading control shown. MW markers are in kDa.

Supplementary Figure 12. Uncut Western blots .

Uncut Western blots for Supplementary Figure 9.

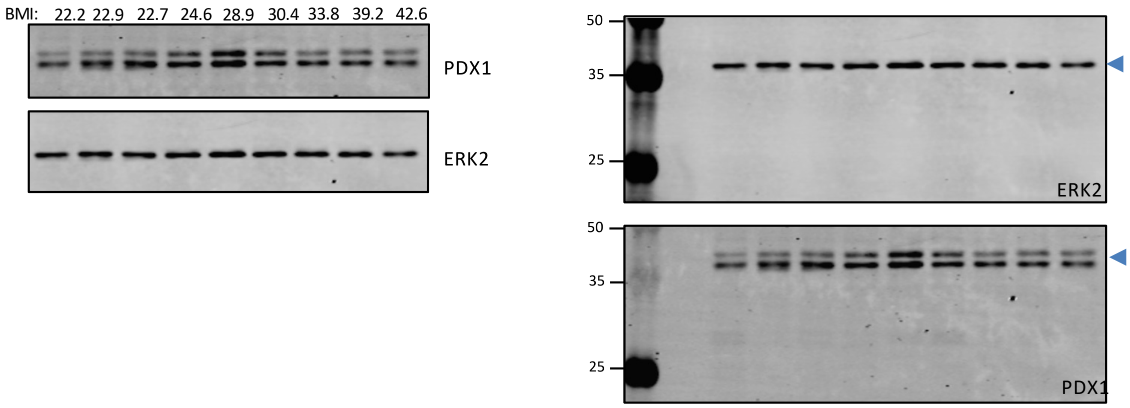

PDX1 blot for human subjects of increasing BMI. Loading control ERK2 is shown. MW markers are in kDa.
