# Supplementary material for: Silencing the G-protein coupled receptor 3-salt inducible kinase 2 pathway promotes human β cell proliferation
Source: Commun Biol. 2021 Jul 23;4:907. doi: 10.1038/s42003-021-02433-2 (PMC8302759; doi:10.1038/s42003-021-02433-2)
Supplement: Supplementary file 7 — Description of Supplementary Files [file 42003_2021_2433_MOESM7_ESM.pdf]

**Description of Additional Supplementary Files**

**File name:** Supplementary Data 1

**Description:** Source data for all figures in manuscript.

**File name:** Supplementary Data 2

**Description:** GPCR LIST with shRNA sequences.

**File name:** Supplementary Data 3

**Description:** Human Donor information.

**File name:** Supplementary Data 4

**Description:** Oligos list.

**File name:** Supplementary Data 5

**Description:** Plasmid list.
