# Supplementary material for: Silencing the G-protein coupled receptor 3-salt inducible kinase 2 pathway promotes human β cell proliferation
Source: Commun Biol. 2021 Jul 23;4:907. doi: 10.1038/s42003-021-02433-2 (PMC8302759; doi:10.1038/s42003-021-02433-2)
Supplement: Supplementary file 8 — Reporting Summary [file 42003_2021_2433_MOESM8_ESM.pdf]

nature research

Corresponding author(s): Robert Screaton

Last updated by author(s): Jun 15, 2021

Reporting Summary

Nature Research wishes to improve the reproducibility of the work that we publish. This form provides structure for consistency and transparency in reporting. For further information on Nature Research policies, see our Editorial Policies and the Editorial Policy Checklist.

Statistics

For all statistical analyses, confirm that the following items are present in the figure legend, table legend, main text, or Methods section.

n/a	Confirmed	
☐	☒	The exact sample size (n) for each experimental group/condition, given as a discrete number and unit of measurement
☐	☒	A statement on whether measurements were taken from distinct samples or whether the same sample was measured repeatedly
☐	☒	The statistical test(s) used AND whether they are one- or two-sided<br>*Only common tests should be described solely by name; describe more complex techniques in the Methods section.*
☐	☒	A description of all covariates tested
☒	☐	A description of any assumptions or corrections, such as tests of normality and adjustment for multiple comparisons
☐	☒	A full description of the statistical parameters including central tendency (e.g. means) or other basic estimates (e.g. regression coefficient) AND variation (e.g. standard deviation) or associated estimates of uncertainty (e.g. confidence intervals)
☐	☒	For null hypothesis testing, the test statistic (e.g. F, t, r) with confidence intervals, effect sizes, degrees of freedom and P value noted<br>*Give P values as exact values whenever suitable.*
☒	☐	For Bayesian analysis, information on the choice of priors and Markov chain Monte Carlo settings
☒	☐	For hierarchical and complex designs, identification of the appropriate level for tests and full reporting of outcomes
☐	☒	Estimates of effect sizes (e.g. Cohen's d, Pearson's r), indicating how they were calculated

Our web collection on statistics for biologists contains articles on many of the points above.

Software and code

Policy information about availability of computer code

Data collection	Western Blots – data collected using the Licor Odyssey CLX<br>Imaging Data – Data collected using the Perkin-Elmer Opera Phenix automated confocal microscope
Data analysis	Western Blots – data analysis using the Image Studio (Version 5.2) from Licor<br>Imaging Data (IF) – Data anaylsed using Columbus high-content imaging analysis software from Perkin Elmer and Harmony software from Perkin Elmer

For manuscripts utilizing custom algorithms or software that are central to the research but not yet described in published literature, software must be made available to editors and reviewers. We strongly encourage code deposition in a community repository (e.g. GitHub). See the Nature Research guidelines for submitting code & software for further information.

Data

Policy information about availability of data

All manuscripts must include a data availability statement. This statement should provide the following information, where applicable:

- Accession codes, unique identifiers, or web links for publicly available datasets
- A list of figures that have associated raw data
- A description of any restrictions on data availability

All data generated or analysed during this study are included in this published article (and its supplementary information files).

Field-specific reporting

Please select the one below that is the best fit for your research. If you are not sure, read the appropriate sections before making your selection.

- [x] Life sciences
- [ ] Behavioural & social sciences
- [ ] Ecological, evolutionary & environmental sciences

For a reference copy of the document with all sections, see nature.com/documents/nr-reporting-summary-flat.pdf

Life sciences study design

All studies must disclose on these points even when the disclosure is negative.

Sample size	Sample sizes were based on previous work.
Data exclusions	No data was excluded from the study.
Replication	For the primary screen, no replication was carried out. It was not possible to source enough islets from a single donor to carry this study out more than once. It is also cost prohibitive. For the secondary screen and all subsequent experiments, the data was obtained successfully from at least three replicates.
Randomization	N/A
Blinding	Investigators were not blinded.

Reporting for specific materials, systems and methods

We require information from authors about some types of materials, experimental systems and methods used in many studies. Here, indicate whether each material, system or method listed is relevant to your study. If you are not sure if a list item applies to your research, read the appropriate section before selecting a response.

Materials & experimental systems

n/a	Involved in the study	
☐	☒	Antibodies
☐	☒	Eukaryotic cell lines
☒	☐	Palaeontology and archaeology
☐	☒	Animals and other organisms
☐	☒	Human research participants
☒	☐	Clinical data
☒	☐	Dual use research of concern

Methods

n/a	Involved in the study	
☒	☐	ChIP-seq
☒	☐	Flow cytometry
☒	☐	MRI-based neuroimaging

Antibodies

Antibodies used

ERK2 (Santa Cruz Biotechnology, sc-1647, clone D-2, Lot number E3019)
p27 (Cell Signaling Technology, 3686, clone D69C12, Lot number 5)
p21(Cell Signaling Technology, 2947, clone 12D1, Lot number 9)
PTEN (Cell Signaling Technology, 9552, Lot number 3)
AKT (Cell Signaling Technology, 2920, clone number 4OD4, Lot number 8)
pAKT Ser473 (Cell Signaling Technology, 4060, clone number D9E, Lot number 24)
p18 (Abcam, ab192239, clone number EPR15891, Lot number GR3193900-3)
PDX1 (Cell Signaling Technology, 5679, clone number D59H3, Lot number 4)
BclXL (Cell Signaling Technology, 2764, clone 54H6, lot number 11)
HSP90 (Santa Cruz Biotechnology, sc-7947, clone H-114)
V5 (Cell Signaling Technology, 13202, Clone D3H8Q, Lot number 6)
SKP2 (Cell Signaling Technology, 2652, clone D3G5, Lot number 3)
p57 (Cell Signaling Technology, 2557, Lot number 2)
p16 INK4a (Cell Signaling Technology, 92803, clone D3W8G, Lot number 1)
p15 INK4b (Abcam, ab53034, Lot number GR79717-30)
p35 (Cell Signaling Technology, 2680, Clone C64B10, Lot number 3)
SIK2 (Cell Signaling Technology, 6919, Clone D28G3, Lot number 2)
C-peptide (DSHB, GN-ID4, Lot numbers 4/27/17-119ug/mL and 2/14/19-217ug/mL)
Insulin (Dako, A0564, Lot number 10125839)
CRTC2
pSer275 CRTC2

Validation	ERK2 (Santa Cruz Biotechnology, sc-1647, clone D-2, Lot number E3019) - literature<br>p27 (Cell Signaling Technology, 3686, clone D69C12, Lot number 5) - literature and gene silencing<br>p21(Cell Signaling Technology, 2947, clone 12D1, Lot number 9) - literature and gene silencing<br>PTEN (Cell Signaling Technology, 9552, Lot number 3) - literature and gene silencing<br>AKT (Cell Signaling Technology, 2920, clone number 4OD4, Lot number 8) - literature<br>pAKT Ser473 (Cell Signaling Technology, 4060, clone number D9E, Lot number 24) - literature<br>p18 (Abcam, ab192239, clone number EPR15891, Lot number GR3193900-3) - literature and gene silencing<br>PDX1 (Cell Signaling Technology, 5679, clone number D59H3, Lot number 4) - literature and gene silencing<br>BclXL (Cell Signaling Technology, 2764, clone 54H6, lot number 11) - literature and gene silencing<br>HSP90 (Santa Cruz Biotechnology, sc-7947, clone H-114) - literature<br>V5 (Cell Signaling Technology, 13202, Clone D3H8Q, Lot number 6) - literature<br>SKP2 (Cell Signaling Technology, 2652, clone D3G5, Lot number 3) - literature<br>p57 (Cell Signaling Technology, 2557, Lot number 2) - literature and gene silencing<br>p16 INK4a (Cell Signaling Technology, 92803, clone D3W8G, Lot number 1) - literature<br>p15 INK4b (Abcam, ab53034, Lot number GR79717-30) - literature<br>p35 (Cell Signaling Technology, 2680, Clone C64B10, Lot number 3) - literature and gene silencing<br>SIK2 (Cell Signaling Technology, 6919, Clone D28G3, Lot number 2) - literature and gene silencing<br>C-peptide (DSHB, GN-ID4, Lot numbers 4/27/17-119ug/mL and 2/14/19-217ug/mL) - literature<br>Insulin (Dako, A0564, Lot number 10125839) - literature<br>CRTC2 - gene silencing<br>pSer275 CRTC2 - experimentally validated in-house

Eukaryotic cell lines

Policy information about cell lines

Cell line source(s)	HEK293T/17 (ATCC, CRL-11268)
Authentication	The cell line was not authenticated.
Mycoplasma contamination	The cell line was not mycoplasma tested.
Commonly misidentified lines (See ICLAC register)	*Name any commonly misidentified cell lines used in the study and provide a rationale for their use.*

Animals and other organisms

Policy information about studies involving animals; ARRIVE guidelines recommended for reporting animal research

Laboratory animals	Mus Musculus, C57Bl/6 background, Male and Female, 6-20 months of age
Wild animals	Study did not involve Wild animals.
Field-collected samples	Study did not involve samples collected from the field.
Ethics oversight	The Comparative Research department from Sunnybrook Research Institute provided approval for the animal study under the AUP-584 number. Approval is based on guidelines provided from Canadian Council of Animal Care (CCAC).

Note that full information on the approval of the study protocol must also be provided in the manuscript.

Human research participants

Policy information about studies involving human research participants

Population characteristics	Human Islets were obtained from cadaver donors that were non-diabetic. Male and female donors were used ranging from 17 to 78 years of age with BMIs ranging from 16.5 to 44.4 kg/m2.
Recruitment	The islets were sourced from the NIDDK-funded Integrated Islet Distribution Program (IIDP islets); University of Alberta, Edmonton, Canada, Clinical Islet Lab of J. Shapiro (Shapiro islets), Alberta Diabetes Institute Research Islet Lab, Canada, lab of P. MacDonald (ADI IsletCore islets), and Toronto University Health Network group, M. Cattral. There was no bias as to receiving these islets as long as they were from non-diabetic donors.
Ethics oversight	We have received an exemption from the Research Ethics Board of Sunnybrook Health Sciences Centre for review of this study. Study # 1540

Note that full information on the approval of the study protocol must also be provided in the manuscript.
